# Sulfur Mediated Interfacial Proton‐Directed Transfer Boosts Electrocatalytic Nitric Oxide Reduction to Ammonia over Dual‐Site Catalysts

**DOI:** 10.1002/anie.202511398

**Published:** 2025-07-09

**Authors:** Zhenlin Wang, Haiyan Duan, Wenqiang Qu, Donglin Han, Xingchi Li, Li Zhu, Xuan Jiang, Danhong Cheng, Yongjie Shen, Ming Xie, Emiliano Cortes, Dengsong Zhang

**Affiliations:** ^1^ International Joint Laboratory of Catalytic Chemistry State Key Laboratory of Advanced Special Steel Innovation Institute of Carbon Neutrality Department of Chemistry College of Sciences Shanghai University Shanghai 200444 P.R. China; ^2^ Nanoinstitute Munich Faculty of Physics Ludwig‐Maximilians‐Universität (LMU) Munich 80539 Germany; ^3^ Department of Chemistry University of Toronto 80 St. George Street Toronto Ontario M5S 3H6 Canada; ^4^ Institute for Chemical Reaction Design and Discovery (WPI‐ICReDD) Hokkaido University Sapporo 001‐0021 Japan; ^5^ Department of Chemical Engineering University of Bath Bath BA2 7AY UK

**Keywords:** Ammonia synthesis, Dual‐site catalysts, Electrocatalysis, Nitric oxide reduction, Sulfur‐mediated

## Abstract

Electrocatalytic nitric oxide reduction reaction (NORR) for ammonia (NH_3_) synthesis represents a sustainable strategy that simultaneously realizes the nitrogen cycle and resource integration. The key issue hindering the NORR efficiency is accelerating proton (*H) transfer to facilitate NO hydrogenation while inhibiting the hydrogen evolution reaction (HER). Herein, we demonstrate an interface‐engineered sulfur‐mediated Cu@Co electrocatalyst (S‐Cu@Co/C) that boosts NORR performance through dual modulation of electronic structure and proton transfer on active sites. A comprehensive program of experimental and theoretical calculations was employed to discover that sulfur incorporation induces electron redistribution in the Cu–Co interface, creating electron‐rich sulfur and electron‐deficient metals. This electronic configuration synergistically enhances NO adsorption on Cu sites and promotes water dissociation on Co sites. More critically, sulfur could direct the rapid transfer of *H from Co to Cu sites, thereby accelerating the NO hydrogenation and suppressing HER. Consequently, S‐Cu@Co/C achieves an NH_3_ yield rate of 655.3 µmol h^−1^ cm^−2^ in a flow cell and a Faradaic efficiency of 92.4% in an H‐cell. Remarkably, the catalyst could maintain continuous electrolysis tests and steady NH_3_ yield up to 100 h. This work provides innovative insights into the fabrication of efficient electrocatalysts via heteroatom‐mediated interfacial engineering strategies.

## Graphical abstract

Sulfur‐mediated electron redistribution and proton‐directed transfer at the Cu‐Co interface significantly boost electrocatalytic NO reduction to NH_3_ over the S‐Cu@Co/C catalyst. The changes in the electronic structure of Cu and Co promote NO adsorption and water dissociation. Meanwhile, sulfur accelerates the transfer of protons generated at Co sites to Cu sites, thereby facilitating NO hydrogenation and suppressing HER.

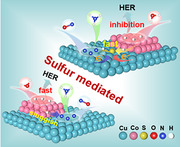

## Introduction

Ammonia (NH_3_) serves as an indispensable chemical precursor for agricultural, pharmaceutical manufacturing, and hydrogen storage systems.^[^ ^1^, ^2^, ^3^ ^]^ Unfortunately, the industrial Haber–Bosch method for NH_3_ synthesis is conducted under harsh conditions, resulting in intensive energy consumption and high‐carbon emissions.^[^ ^4^, ^5^, ^6^ ^]^ Electrocatalytic nitrogen reduction reaction (NRR) as a novel technology for ambient‐condition NH_3_ synthesis,^[^ ^7^ ^]^ while its practical application remains limited by the low Faradaic efficiency (FE) and NH_3_ yield rate due to the inherent inertness of N≡N (945 kJ mol^−1^) and competitive hydrogen evolution reaction (HER).^[^ ^8^ ^]^ In contrast, electrocatalytic nitric oxide reduction reaction (NORR) possesses dual advantages of thermodynamic favorability (NO bond energy of 204 kJ mol^−1^) and elimination of NO pollution from the flue gas.^[^ ^9^, ^10^ ^]^ Nevertheless, the NORR involves a complex multi‐electron and proton (*H) coupled process,^[^ ^11^ ^]^ where the kinetic bottlenecks arise from the deoxygenation and hydrogenation steps, while the FE is influenced by the HER.^[^ ^12^ ^]^ Therefore, engineering of the electrocatalytic interface to accelerate proton transfer and utilization is critical for simultaneously achieving superior NH_3_ yield rate and FE.

Previous studies have established that copper (Cu) is an excellent NORR catalyst due to its exceptional NO activation capability, yet the performance remains fundamentally constrained by the insufficient supply of *H.^[^ ^13^, ^14^ ^]^ Strategic integration with cobalt (Co), which features remarkable H_2_O dissociation capability,^[^ ^15^, ^16^ ^]^ partially addresses this limitation but introduces a competitive HER at Co sites.^[^ ^17^ ^]^ This dichotomy results in an unsatisfactory NH_3_ yield rate and FE in conventional Cu@Co architectures. Optimizing the transfer and utilization of *H between the two motifs is anticipated to inhibit H_2_ production and accelerate NO hydrogenation.^[^ ^18^ ^]^ Crucially, the interface on biphasic structured catalysts with variable electronic and geometrical structures offers distinctive pathways for facilitating proton transfer.^[^ ^19^, ^20^ ^]^ From this perspective, precision modification of the Cu@Co interface to accelerate *H transfer while modulating the electronic structure at the reaction sites to expedite the hydrogenation rate is a distinctive prospective strategy to boost the NORR efficiency.

Herein, we propose a Cu–Co biphasic electrocatalyst with atomic‐level interface engineering via sulfur mediation (S‐Cu@Co/C), thus enabling the dual function of establishing a channel for rapid *H transfer between the Cu‐Co interface and modulating electron distribution on the active centers. The sulfur is stabilized on Cu sites and induces a redistribution of electrons at the Cu‐Co interface, forming electron‐rich sulfur and electron‐deficient metal centers. Consequently, the Cu sites enhance NO adsorption, and adjacent Co sites promote water dissociation, while sulfur could function as an effective mediator to facilitate the rapid *H transfer from Co to Cu sites, thereby accelerating the hydrogenation pathway and suppressing H_2_ generation. Electrocatalytic NORR performance evaluations confirm the superior NORR performance of the S‐Cu@Co/C catalyst, achieving an NH_3_ yield rate of 439.73 µmol cm^−2^ h^−1^ with a FE of 92.4% at−0.6 V vs. reversible hydrogen electrode (RHE) in H‐cell. The NH_3_ yield rate was boosted up to 655.3 µmol cm^−2^ h^−1^ in the flow cell and enabling a long‐time electrolysis test of 100 h with steady NH_3_ yield. A combination of density functional theory (DFT) calculations and quasi‐in situ electron paramagnetic resonance (EPR) spectroscopy explicitly elucidates the mechanism of enhanced activity triggered by sulfur‐mediated interfacial electronic modulation and rapid *H transfer. In situ spectroscopy monitored the complete reaction pathway from NO adsorption to NH_3_ generation, confirming the critical role of sulfur in coordinating sequential proton transfer. This pioneering approach constitutes a novel strategy for catalytic interface engineering through heteroatom mediation, opening up the possibility of designing more efficient NORR electrocatalysts.

## Results and Discussion

**Figure 1:**
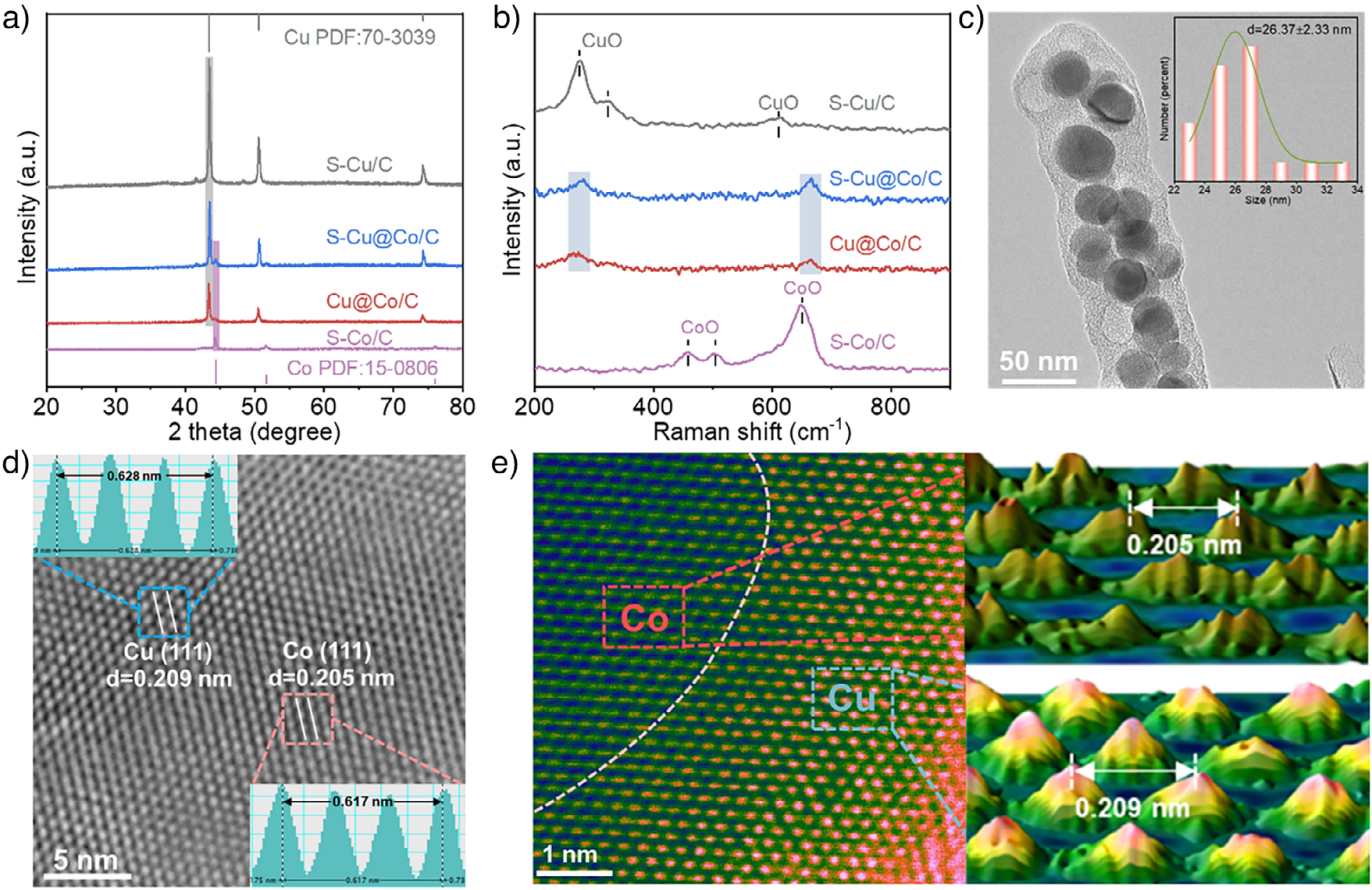
Morphology and structure characterization. a) XRD patterns of the catalysts; b) Raman spectra of the catalysts. c,d) HRTEM image of S‐Cu@Co/C catalyst. The inset in (c) is the particle size distribution, and in (d) is the calculated lattice fringes distance. e) AC‐HAADF‐STEM image of S‐Cu@Co/C catalyst and the three‐dimensional atomic topology image of the marked region.

The S‐Cu@Co/C catalyst was obtained through a two‐step fabrication strategy, as schematically illustrated in Figure S1. Initially, a hollow rod‐shaped metal‐organic framework precursor was synthesized via the solvothermal method (Figure S2), followed by pyrolysis under a N_2_ atmosphere to yield the final catalysts. The gradient calcination of precursors (300 to 800 °C) revealed a small amount of sulfide at 300 °C. Subsequent heating to 700 °C triggered Co phase emergence, while further elevation to 800 °C made bimetallic phases more distinct (Figure S3). To investigate the evolution of sulfur species in the catalyst, a comparison catalyst was prepared by the same method without adding 1,3,5‐benzenetricarboxylic acid (H_3_BTC). The X‐ray diffraction (XRD) results revealed the catalyst to be sulfide (Figure S4), which is completely different from the distinct biphasic Cu (PDF#70–3039) and Co (PDF#15–0806) crystalline character of S‐Cu@Co/C and Cu@Co/C (Figure 1a). Therefore, the comparative experiment confirmed that H_3_BTC, as a carbon source, can gradually reduce Cu and Co species to the metallic state during calcination. Raman spectra analysis identified the presence of partially surface‐oxidized species in S‐Cu/C and S‐Co/C^[^ ^21^ ^]^ (Figure 1b). However, the signals of these species were attenuated in S‐Cu@Co/C and Cu@Co/C, indicating that Cu and Co are mainly present as metallic forms.

High‐resolution transmission electron microscopy (HRTEM) showed that S‐Cu@Co/C and Cu@Co/C all exhibited carbon‐supported nanoparticle structures (Figures 1c and S5), in contrast to the morphology of the catalyst prepared without H_3_BTC (Figure S6). The Raman spectra of S‐Cu@Co/C and Cu@Co/C demonstrated the presence of characteristic G‐band and D‐band peaks (Figure S7), validating the carbon existence. The specific surface area and pore structure of the catalysts confirmed that S‐Cu@Co/C has a larger specific surface area (162 m^2^ g^−1^) and more abundant pore structure than Cu@Co/C, and the pore sizes range from 1.5 to 7 nm (Figures S8 and S9). This abundant pore structure originates from the formation of porous carbon on the catalysts. The transmission electron microscopy‐energy dispersive spectrometer (EDS) mapping and inductively coupled plasma optical emission spectrometer (ICP‐OES) provided insights into the elemental distribution and composition (Figures S10,11 and Table S1). The results show that Co content was significantly lower than Cu, while the sulfur was uniformly distributed across the metals. The presence of oxygen around the metals originates from the exposure of the catalyst to air, where the metal was partially oxidized. The distinct lattice fringes distance in the HRTEM image of S‐Cu@Co/C was measured to be 0.209  and 0.205 nm (Figure 1d), corresponding to the Cu (111) and Co (111) planes, respectively. This structural characterization was further elaborated by aberration‐corrected HAADF‐STEM (AC‐HAADF‐STEM) image (Figure 1e), which shows a clear interfacial structure between Cu and Co in S‐Cu@Co/C. Therefore, the above characterizations indicate the successful fabrication of S‐mediated Cu@Co/C biphasic catalysts with an engineered interfacial structure.

**Figure 2:**
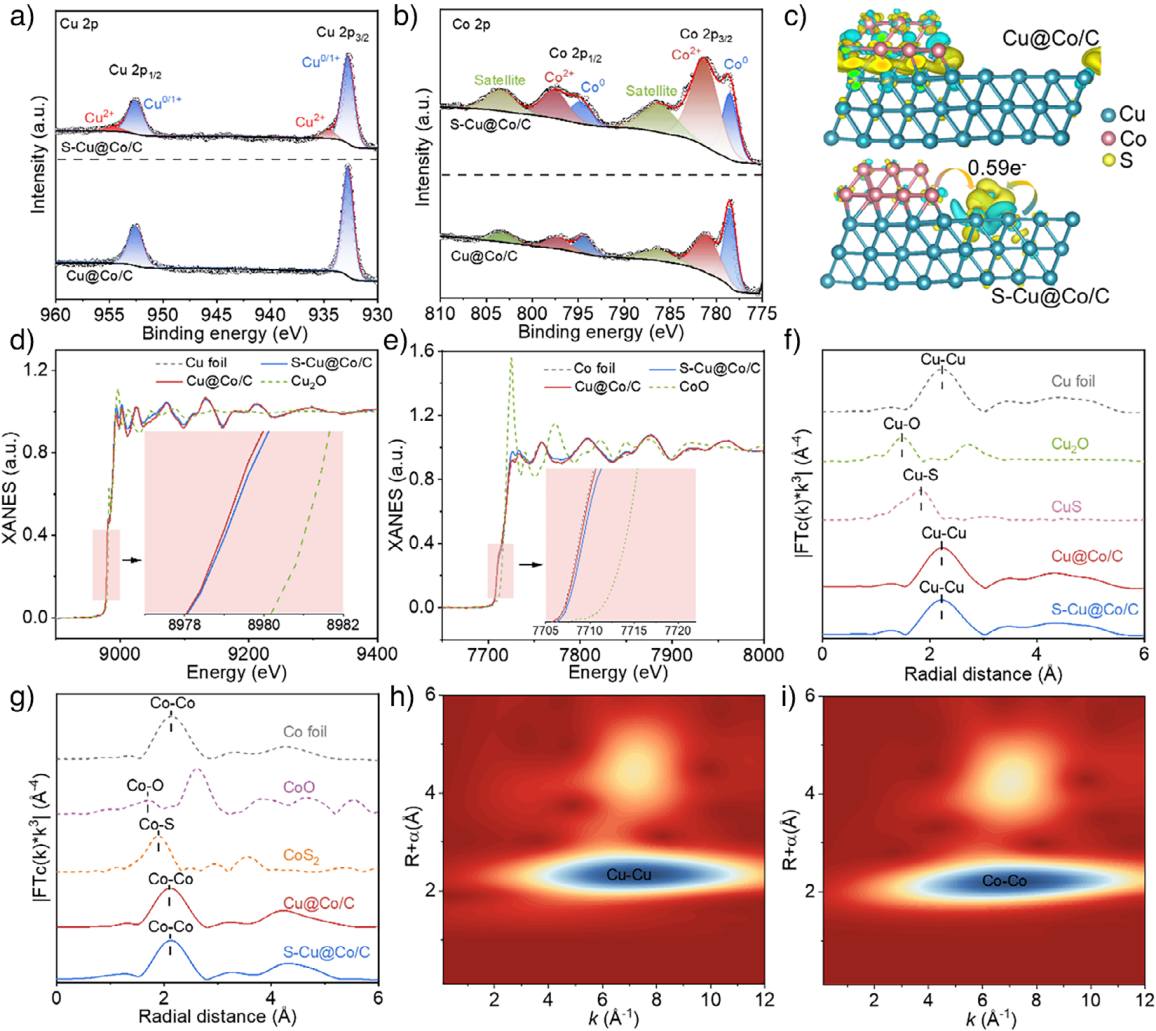
Characterizations of catalysts. a) Cu 2p and b) Co 2p XPS spectra of S‐Cu@Co/C and Cu@Co/C; c) The charge density difference analysis for S‐Cu@Co/C and Cu@Co/C (yellow and cyan colors represent electron accumulation and depletion regions, respectively); d) Cu K‐edge XANES spectra of S‐Cu@Co/C and Cu@Co/C. The inset is the enlarged pre‐edge region; e) Co K‐edge XANES spectra of S‐Cu@Co/C and Cu@Co/C. The inset is the enlarged pre‐edge region; f) The FT‐EXAFS spectra of Cu foil, Cu_2_O, CuS, S‐Cu@Co/C and Cu@Co/C; g) The FT‐EXAFS spectra of Co foil, CoO, CoS_2_, S‐Cu@Co/C and Cu@Co/C; (h‐i) WT contour plots of Cu and Co K‐edge EXAFS signals of S‐Cu@Co/C.

X‐ray photoelectron spectroscopy (XPS) and X‐ray absorption near‐edge spectra (XANES) are employed to investigate the valence state information of the prepared catalysts. For the Cu 2p XPS scan (Figure 2a), it can be found that a slight amount of Cu^2+^ appeared in S‐Cu@Co/C compared to Cu@Co/C, indicating the electron loss on Cu. In the Co 2p XPS scan (Figure 2b), the proportion of Co^0^ in S‐Cu@Co/C was decreased, while Co^2+^ was increased compared to Cu@Co/C, suggesting that the Co also loses electrons in S‐Cu@Co/C. As a consequence, electrons will transfer from the metals to sulfur, creating an electron‐rich sulfur and electron‐deficient metals. To verify this phenomenon, the charge density difference analysis of the two catalysts was conducted by DFT calculations. Figure 2c shows electron transfer between Cu and Co in the Cu@Co/C. However, the incorporated sulfur in the S‐Cu@Co/C could attract electrons from the metals (Δq = 0.59 e^−^), consistent with the XPS tests. Meanwhile, the S 2p XPS scan verified the presence of S^2−^ and revealed that the sulfur could be bonded to the Cu (Figure S12). Furthermore, the pre‐edge curves of Cu in S‐Cu@Co/C and Cu@Co/C are all located close to Cu foil (Figure 2d), implying that the valence state of Cu in S‐Cu@Co/C and Cu@Co/C is both close to metallic Cu. Additionally, in the Cu K‐edge, the pre‐edge curve of S‐Cu@Co/C displayed a positive energy shift relative to Cu@Co/C, suggesting that sulfur mediation induced partial electron depletion on Cu sites.^[^ ^22^ ^]^ For the Co K‐edge (Figure 2e), the pre‐edge curves for both catalysts are located between Co foil and CoO, implying a higher valence state of Co in the catalysts relative to metallic Co. Furthermore, the pre‐edge curve in Co K‐edge of S‐Cu@Co/C also exhibited a positive shift compared to Cu@Co/C, consistent with the valence state increase observed in Co. These results indicate that sulfur mediates the alteration of Cu and Co electronic structure, corroborating the above XPS results.

Extended X‐ray absorption fine structure (EXAFS) is introduced to investigate the coordination structure of the metals in the catalysts. A major peak at 2.2Å is found in the Fourier‐transformed k^3^‐weighted spectra of the Cu K‐edge in the R space of S‐Cu@Co/C and Cu@Co/C (Figure 2f), corresponding to Cu─Cu bonding and agreement with the Cu foil.^[^ ^23^ ^]^ In the Co K‐edge of S‐Cu@Co/C and Cu@Co/C (Figure 2g), the major peak at 2.1 Å in the R space assigned to the Co─Co bond coincides with the Co foil.^[^ ^24^ ^]^ The coordination bond of metals with carbon or nitrogen was not observed, reconfirming that Cu and Co in the S‐Cu@Co/C mainly exist in the form of Cu─Cu and Co─Co, which are highly compatible with the wavelet transform (WT) contour plot of the S‐Cu@Co/C (Figure 2h–i). The coordination environment of sulfur with metals was analyzed based on EXAFS fitting (Figure S13,S14 and Table S2). The fitting results suggested that sulfur preferentially binds to Cu with an average coordination number of 0.5. The DFT calculations were also conducted to validate the fitting results and confirmed that the Cu─S bonds are more stable than the Co─S bonds (Figure S15). Thus, sulfur at the Cu─Co interface derives from the small amount of sulfur retained on the Cu surface during the high‐temperature calcination process, forming a stable coordination structure with Cu. Moreover, the cyclic voltammetry test showed that S‐Cu@Co/C did not exhibit obvious redox peaks of sulfide compared to the comparison catalyst, indicating the formation of a stable Cu‐S structure (Figure S16). The above analytical results demonstrate that sulfur mediation is an effective interfacial engineering strategy to modulate the electronic configuration of Cu@Co by targeted charge redistribution.

**Figure 3:**
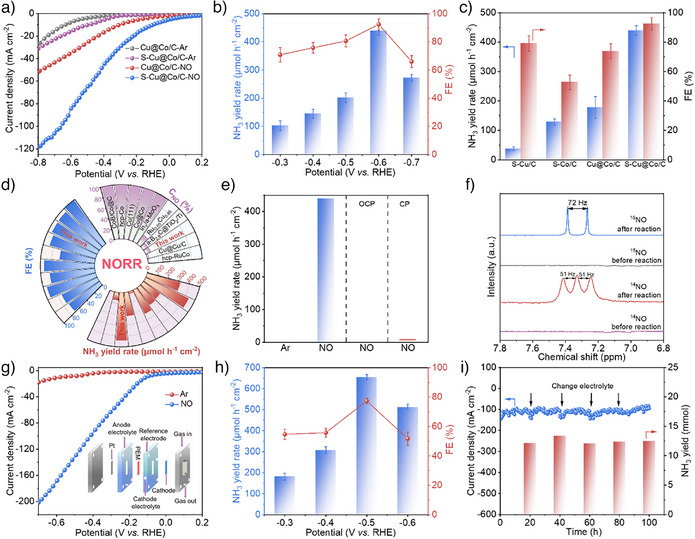
NORR performance. a) LSV curves of prepared catalysts under NO and Ar in H‐cell; b) NH_3_ yield rate and FE over S‐Cu@Co/C at series potential; c) NH_3_ yield rate and FE over prepared catalysts at −0.6 V vs. RHE in H‐cell; d) Comparison of the NORR performance of S‐Cu@Co/C with the reported catalysts; e) NORR performance of S‐Cu@Co/C under series conditions (OCP and CP represent open circuit potential and carbon paper); f) NMR spectra of isotope labeling experiments on S‐Cu@Co/C; g) LSV curves of S‐Cu@Co/C under NO and Ar in flow cell; h) NH_3_ yield rate and FE over S‐Cu@Co/C in flow cell; i) Current density and NH_3_ yield of S‐Cu@Co/C for stability test of NORR in flow cell at −0.5 V vs. RHE. All tests were conducted in PBS electrolytes. (Error bars mean the standard deviations of three independent experiments, the center value of the error bars means the average of three independent experiments).

The electrocatalytic NORR performance of the prepared catalysts was evaluated by a gas‐tight H‐type cell with PBS electrolyte (Phosphate Buffer Saline). The linear sweep voltammetry (LSV) tests in Ar‐saturated electrolyte reveal a consistently higher current density on S‐Cu@Co/C than on Cu@Co/C at identical potentials, indicating a faster water dissociation on S‐Cu@Co/C (Figure 3a). Furthermore, a remarkable enhancement of the current density was recorded when Ar was switched to NO over S‐Cu@Co/C and Cu@Co/C catalysts in the lsv curves, implying that NO has been effectively reduced on the catalysts. The current density of S‐Cu@Co/C is 2.3 times higher compared to that of Cu@Co/C, suggesting that sulfur promotes the NORR process. After 1 h of chronoamperometry (CA) test, the NH_3_ yield rate and FE were quantified by colorimetric methods (Figure S17). The effects of different metals and sulfur content on NORR were first investigated. The results suggested that the ratio of Cu to Co was 3:1, and the amount of added sulfur feedstock was 0.3, which presented the most outstanding NORR performance (Figure S18).

Figure 3b shows a volcanic trend in the NORR performance of S‐Cu@Co/C at series potentials. When the potential increases from ‐0.3 to −0.6 V vs. RHE, NO activation and hydrogenation rates accelerate, resulting in a gradual rise in NH_3_ yield rate and FE. However, when the applied potential exceeds −0.6 V vs. RHE, the NH_3_ yield rate and FE declined due to the competitive HER. The highest NH_3_ yield rate of 439.73 µmol h^−1^ cm^−2^ (0.440 mol h^−1^ g_cat_^−1^, Table S3) and FE of 92.4% were achieved at ‐0.6 V vs. RHE (Figure S19). The NH_3_ yield rate is 2.4 times higher than that of Cu@Co/C and significantly exceeds that of S‐Cu/C and S‐Co/C, respectively (Figure 3c). More excitingly, this performance obtained at lower potential and NO concentrations is excellent compared to most currently reported NORR systems (Figure 3d and Table S4). Instead, the catalyst prepared without H_3_BTC only achieved an NH_3_ yield rate of 69.41 µmol h^−1^ cm^−2^ and FE of 58.15% at ‐0.6 V vs. RHE (Figure S20), corroborating that sulfide catalysts have much lower NORR performance than sulfur‐mediated catalysts. The larger double‐layer capacitance (C_dl_) (17.71 mF·cm^−2^) and smaller charge transfer resistance (0.65 Ω) of S‐Cu@Co/C compared to Cu@Co/C suggest higher intrinsic activity and faster electron transfer during the NORR, which is favorable for electrocatalytic NO reduction (Figures S21 and S22). Additionally, the large specific surface area and appropriate pore structure of S‐Cu@Co/C could facilitate mass transfer in the NORR. The NORR performance of the S‐Cu@Co/C was also tested in an alkaline electrolyte (1 M KOH), which revealed a significant decline in NORR efficiency, suggesting that alkaline conditions are unfavorable for the NO reduction (Figure S23).

To probe the origin of the produced NH_3_, NORR measurements under different conditions were conducted. The NH_3_ was not observed under an Ar atmosphere with applied potential and open‐circuit condition with NO flow, also with negligible NH_3_ accumulation on blank carbon paper (Figure 3e). Simultaneously, NO_3_^−^ and NO_2_^−^ were not detected in the electrolyte after electrolysis (Figure S24). Furthermore, the ^15 ^N isotope labeling experiment was conducted, and the product NH_3_ was analyzed by the ^1^H nuclear magnetic resonance (NMR) spectra (Figure 3f). The NH_3_ obtained when ^14^NO as the reactant showed a triple characteristic peak, whereas the NH_3_ presented a double characteristic peak when ^15^NO was used as the reactant,^[^ ^25^ ^]^ which unambiguously verified the NO‐derived origin of the NH_3_ products. These results firmly validate that the NH_3_ produced originates from NO reduction rather than an external nitrogen source. The by‐products during NORR, including H_2_, N_2_, N_2_O, N_2_H_4,_ and NH_2_OH were monitored by online differential electrochemical mass spectrometry (DEMS) and colorimetric methods. The DEMS analysis identified H_2_ as the gas by‐product (Figure S25), while colorimetric methods confirmed that there were no N_2_H_4_ and NH_2_OH (Figures S26 and S27). In contrast, comparing the H_2_ signal reveals a substantial enhancement of HER on Cu@Co/C relative to S‐Cu@Co/C (Figure S28), revealing sulfur‐mediated suppression of HER on S‐Cu@Co/C. The quantitative analysis by gas chromatography (GC) also revealed that the lower FE of H_2_ on S‐Cu@Co/C (6.17%) relative to Cu@Co/C (24.3%) (Figure S29). In addition to exceptional performance, S‐Cu@Co/C also demonstrated remarkable cyclic stability, with no significant decrease in NORR performance after 15 continuous electrolysis tests, confirming robust structural stability under continuous electrochemical operation (Figure S30 and S31).

The NO electroreduction performance of S‐Cu@Co/C was also conducted with gas diffusion electrodes (GDE) using the flow cell. Figure 3g shows that the current density of S‐Cu@Co/C in the flow cell was increased to 200 mA cm^−2^ in comparison with the 103 mA cm^−2^ in the H‐cell. Remarkably, the NH_3_ yield rate reached 655.3 µmol h^−1^ cm^−2^ at −0.5 V vs. RHE, representing a tremendous enhancement compared with the H‐cell and approaching the state‐of‐the‐art performance reported. The results were also confirmed by NMR spectroscopy, and it was comparable to the colorimetric method (Figures S32 and S33). In addition, the N_2_H_4_ and NH_2_OH were not detected in the flow cell tests (Figure S34). Therefore, the decrease in FE in the flow cell test is due to the lower solubility of NO (1.92 mmol L^−1^ atm^−1^, at 25 °C),^[^ ^26^ ^]^ leading to increased water‐catalyst contact frequency, and enhanced water reduction. Moreover, the flowing electrolyte promotes the desorption of gas bubbles on the catalyst surface and accelerates the H_2_ generation. The operational durability of electrocatalysts constitutes a critical performance metric for practical application. As shown in Figure 3i, the S‐Cu@Co/C exhibited 100 h of continuous electrolysis in the flow cell at −0.5 V vs. RHE while maintaining a constant NH_3_ yield. The crystal and morphological structure of S‐Cu@Co/C were not destroyed, and the surface valence state remained unchanged after the reaction (Figures S35–37), evidencing exceptional structural integrity over the long‐time test. These electrochemical and structural stability metrics substantiate the feasibility of S‐Cu@Co/C for sustainable electrocatalytic NO to NH_3_. Thus, to assess the application prospects of S‐Cu@Co/C, a Zn‐NO battery system was constructed with catalyst as the cathode. The results show that the assembled cells exhibit a power density of 14.23 mW cm^−2^ and an exceptional NH_3_ yield of 3192.6 µg h^−1^ mg_cat_^−1^, surpassing most reported catalysts in both energy output and NH_3_ production efficiency (Figure S38 and Table S5).

**Figure 4:**
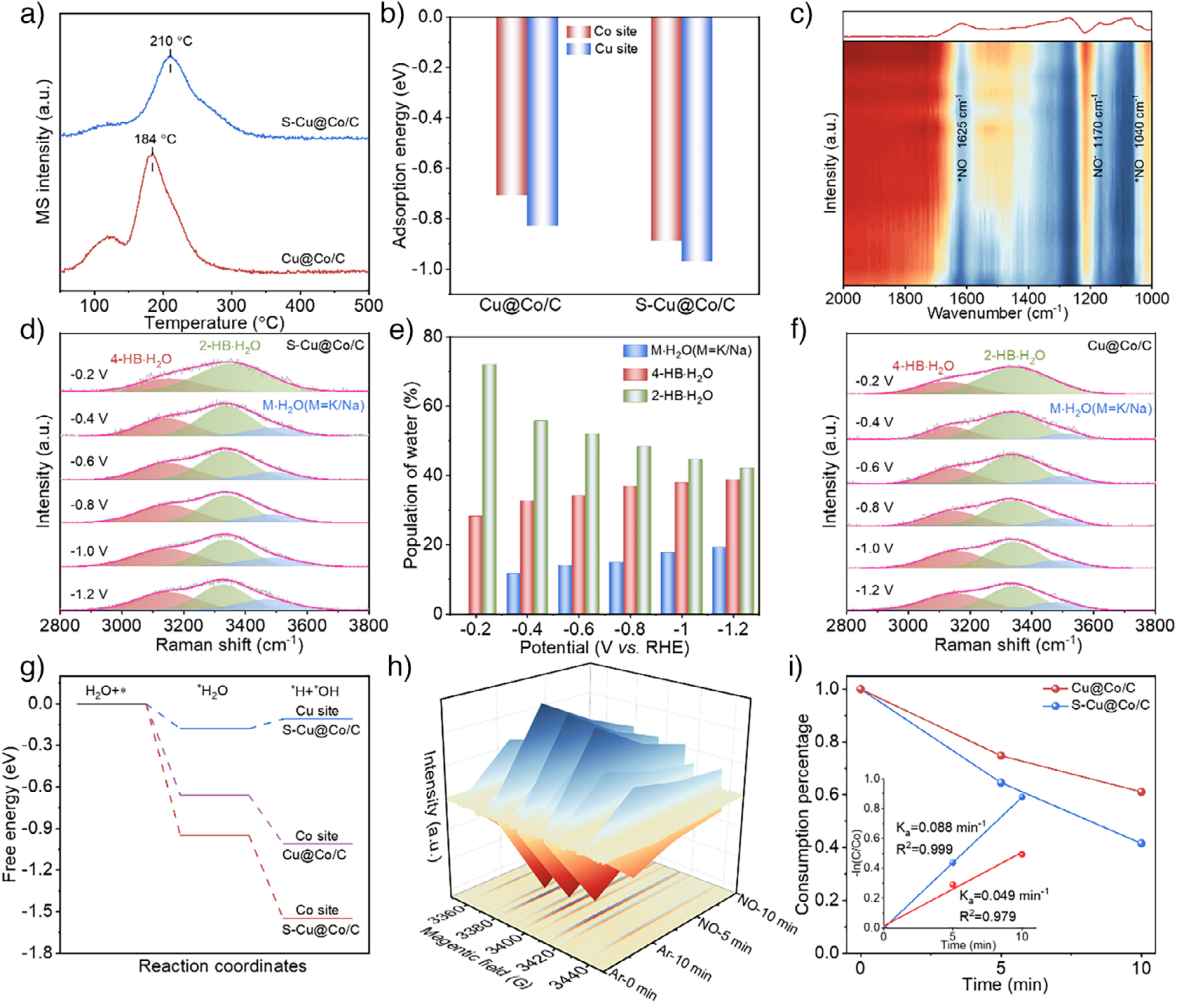
NO adsorption and water dissociation investigations. a) NO‐TPD of S‐Cu@Co/C and Cu@Co/C; b) Calculated NO adsorption energy on Cu and Co sites in S‐Cu@Co/C and Cu@Co/C; c) Operando ATR‐IRAS spectra of NO adsorption on S‐Cu@Co/C without applying bias potential in PBS electrolyte; d) Gaussian‐fitted peaks of in situ Raman spectra of S‐Cu@Co/C; e) Population of interfacial water of S‐Cu@Co/C; f) Gaussian‐fitted peaks of in situ Raman spectra of Cu@Co/C; g) Calculated water dissociation energy on Cu and Co sites in S‐Cu@Co/C and Cu@Co/C; h) Quasi‐in situ electrochemical EPR signals for S‐Cu@Co/C in PBS electrolyte at −0.6 V vs. RHE; i) Comparison of ·H signal degradation rates on S‐Cu@Co/C and Cu@Co/C.

To elucidate the mechanistic origins of the sulfur‐mediated catalytic process, a series of in situ and quasi‐in situ tests were employed to explore the differences in NO adsorption and water dissociation. The NO temperature programmed desorption (NO‐TPD) tests show that S‐Cu@Co/C exhibits stronger NO adsorption than Cu@Co/C, indicating that sulfur induces enhanced adsorption of NO on the metals (Figure 4a). Moreover, S‐Cu/C presents strengthened adsorption compared to S‐Co/C, suggesting that NO prefers to adsorb on Cu sites (Figure S39). The DFT calculations also demonstrated that the adsorption energy of NO on the Cu sites (−0.83 eV) is higher than on the Co sites (−0.71 eV) and that sulfur mediation strengthens the adsorption of NO on the Cu sites (−0.97 eV) (Figure 4b). Correspondingly, Operando attenuated total reflection infrared absorption spectroscopy (ATR‐IRAS) revealed the presence of NO bands (1040 cm^−1^ and 1625 cm^−1^) and NO^−^ (1170 cm^−1^) species on the S‐Cu@Co/C surface when NO was fed under the open‐circuit potential condition (Figure 4c).^[^ ^27^ ^]^ These results confirm that the adsorption of NO mainly occurs on Cu sites and that sulfur‐mediated interfacial structure could enhance the NO adsorption. The interfacial water adsorbed on the catalyst surface under neutral or alkaline electrolyte is the primary proton source for NO hydrogenation.^[^ ^28^ ^]^ In situ Raman spectroscopy was employed to investigate the interfacial water on the catalyst surface.^[^ ^29^ ^]^ The broad peak was located at 3000–3800 cm^−1^ could be categorized into 4‐HB·H_2_O, 2‐HB·H_2_O, and alkali metalionized water (K/Na·H_2_O) by Gaussian fitting,^[^ ^30^ ^]^ respectively (Figure 4d). Among them, the proportion of K/Na·H_2_O represents the strength of water dissociation on the catalyst surface.^[^ ^31^ ^]^ The proportion of K/Na·H_2_O on S‐Cu@Co/C increases to 19.2% with increasing potential (Figure 4e), surpassing the 13.9% obtained on Cu@Co/C (Figures 4f and S40). Therefore, the dissociation of water to supply *H is more favorable on S‐Cu@Co/C. To elucidate the active sites governing this process, DFT calculations disclosed that Co sites are more favorable for water dissociation relative to Cu sites. Importantly, sulfur incorporation accelerates the water dissociation on the Co sites, which is consistent with the Raman results (Figures 4 g and S41). Moreover, the sulfur site is incapable of water dissociation processes (Figure S42). From the above analysis, sulfur mediation successfully strengthened NO adsorption and stimulated water dissociation on S‐Cu@Co/C, establishing fundamental prerequisites for achieving efficient NORR.

Furthermore, quasi‐in situ EPR tests were employed to monitor *H evolution during the NORR process over S‐Cu@Co/C and Cu@Co/C catalysts. The ·H can be effectively trapped by 5,5‐dimethyl‐1‐pyrroline‐N‐oxide (DMPO) and displays nine characteristic peaks. Both catalysts reached maximum ·H intensity under Ar gas saturation electrolyte at −0.6 V vs. RHE after 10 min, and the ·H signal intensity on S‐Cu@Co/C was higher than on Cu@Co/C, reconfirming that water dissociation was much favorable on S‐Cu@Co/C (Figures 4 h and S43). Notably, the signals decrease dramatically when switched to NO, signifying that ·H was consumed through NO hydrogenation pathways. The decay kinetic constants of the ·H were calculated using the first‐order kinetic equation. Strikingly, the kinetic constant on S‐Cu@Co/C is considerably higher than that of Cu@Co/C (Figure 4i), directly evidencing sulfur‐mediated enhancement of *H utilization efficiency for NO hydrogenation. The reversible recovery of the ·H signal observed upon Ar reintroduction further establishes that the regulation of proton utilization is crucial for controlling the NORR efficiency (Figure S44). In addition, when tert‐butyl alcohol (TBA) was added to the NORR process, the NH_3_ yield rate and FE of S‐Cu@Co/C declined to 85.74 µmol h^−1^ cm^−2^ and 61.98%, reconfirming the vitally important role of hydrogen for NO hydrogenation (Figure S45). Integrating the aforementioned in situ experiments and calculation results, it could be concluded that sulfur mediation enhances NO adsorption on Cu sites and accelerates water dissociation on Co sites. More significantly, sulfur incorporation accelerates the proton transfer and utilization, thereby kinetically favoring the NO hydrogenation.

**Figure 5:**
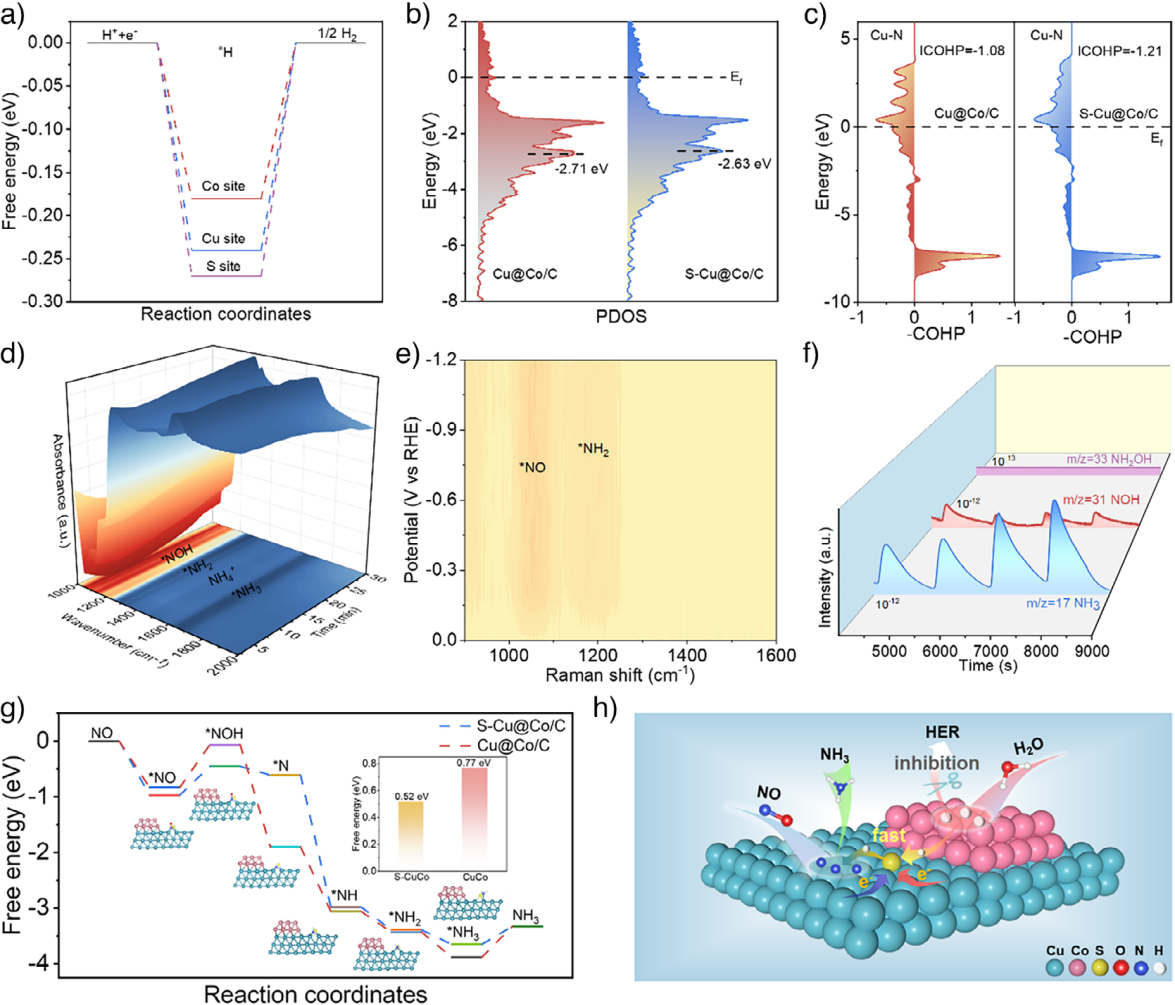
Reaction pathway and mechanism. a) The Gibbs free energy diagram of *H adsorption on S, Cu and Co sites in S‐Cu@Co/C; b) The calculated PDOS of S‐Cu@Co/C and Cu@Co/C; c) The COHP of *NO on S‐Cu@Co/C and Cu@Co/C; d) Operando ATR‐IRAS spectra of electrocatalytic NORR under − 0.6 V vs. RHE over S‐Cu@Co/C; e) In situ Raman spectra of electrocatalytic NORR under − 0.6 V vs. RHE over S‐Cu@Co/C; f) DEMS spectra of NORR over S‐Cu@Co/C; g) The Gibbs free energy diagram of the NORR over S‐Cu@Co/C and Cu@Co/C. The insets are the diagram of the adsorption of intermediates on S‐Cu@Co/C after structural optimization and the ΔG of the RDS; h) Schematic of NORR mechanism over S‐Cu@Co/C.

To elucidate the detailed *H transfer pathway mediated by sulfur, DFT calculations were employed to compare the Gibbs free energies of *H adsorption (ΔG_*H_) on sulfur, Cu and Co sites in S‐Cu@Co/C. The results show that Co exhibits the weakest proton adsorption (−0.18 eV) and favors proton desorption, while sulfur displays the strongest proton adsorption (−0.27 eV), and Cu shows intermediate affinity (−0.24 eV) (Figure 5a). Furthermore, calculations of proton transfer energy barrier also suggest that the ΔG from the Co site to the sulfur site is lower than that to the Cu site, confirming that sulfur could mediate proton transfer (Figure S46). Overall, upon water dissociation at the Co site, the protons undergo desorption and subsequent adsorption on sulfur sites. These protons rapidly migrate through the sulfur sites and efficiently transfer to the adjacent Cu sites to participate in the NO hydrogenation. Moreover, the strong adsorption of *H at the sulfur sites effectively suppresses H_2_ evolution at the Co sites. In addition, projected density of state (PDOS) calculations revealed that sulfur induced the d‐band center of S‐Cu@Co/C shifted toward the Fermi level, accounting for the strengthened reactants adsorption through optimized electronic structure^[^ ^32^ ^]^ (Figure 5b). The crystal orbital Hamilton population (COHP) further suggests that sulfur results in the Cu─N bond formed by NO adsorption with a larger ICOHP value (Figure 5c), thereby confirming the reinforced NO adsorption on S‐Cu@Co/C. The PDOS calculations additionally prove the effective activation of NO on S‐Cu@Co/C, where the metal d‐orbitals and the bonding and antibonding orbitals of NO are significantly hybridized (Figure S47). To uncover the NORR pathway on the catalysts, the Operando ATR‐IRAS tests were conducted to characterize the generation of reaction intermediates. As illustrated in Figure 5d, the signals of *NOH (1120 cm^−1^) and *NH_2_ (1330 cm^−1^) intermediates gradually increase with increasing reaction time at −0.6 V vs. RHE on S‐Cu@Co/C, accompanied by characteristic peaks for generated NH_4_^+^ and NH_3_ corresponding to 1480 and 1654 cm^−1[^ ^33^ ^]^ respectively. Meanwhile, no signal was monitored for *NHO reaction intermediate. In contrast, although similar intermediates and products were monitored on Cu@Co/C, the intensity of the peaks was significantly weakened compared to S‐Cu@Co/C (Figure S48). In addition, in situ Raman spectra tested from OCP to −0.6 V vs. RHE revealed two prominent peaks coinciding with NO adsorption and the formation of *NH_2_ intermediate on S‐Cu@Co/C (Figure 5e).^[^ ^34^ ^]^ Further validation of these intermediates by online DEMS analysis identified mass signals at *m/z* = 17 and 31 corresponding to NH_3_ and NOH, and no NH_2_OH signal was recorded (Figure 5f). Correspondingly, the DEMS signals of Cu@Co/C are consistent with S‐Cu@Co/C (Figure S49), while the signal intensity of the products and intermediates was lower than that of S‐Cu@Co/C. These observations affirmed that sulfur mediation accelerates the proton transfer and utilization efficiency and facilitates NO hydrogenation without altering the reaction pathway. The DFT calculations indicated that the *ΔG* of *NHO (0.61 eV) on S‐Cu@Co/C is higher than that of *NOH (0.52 eV), suggesting the rate‐determining step (*NO→*NOH) is consistent for both catalysts (Figures 5 g and S50), and the ΔG for Cu@Co/C (0.77 eV) is higher than S‐Cu@Co/C (0.52 eV). This theoretical evidence aligns with experimental observations, indicating that sulfur incorporation facilitates NO hydrogenation at the Cu sites via optimizing proton transfer kinetics. Integrating the above‐mentioned results, Figures 5h illustrates the NORR mechanism on S‐Cu@Co/C. The sulfur at the Cu‐Co biphasic interface induces interfacial electron redistribution, creating electron‐rich sulfur and electron‐deficient metals. This electronic reconstruction enhances the NO adsorption activation on Cu sites and accelerates water dissociation on adjacent Co sites through the optimized electronic states. The generated *H at Co sites is subsequently efficiently transferred to Cu sites via sulfur‐mediated pathway, thereby synergistically lowering the NO hydrogenation barrier while suppressing competing HER. This interfacial charge modulation and proton transport optimization collaboratively boost the effective NORR process.

## Conclusion

In summary, we demonstrate high‐efficiency electrocatalytic NO reduction to NH_3_ through sulfur‐mediated interfacial engineering in a Cu‐Co biphasic electrocatalyst (S‐Cu@Co/C). The optimized S‐Cu@Co/C catalyst exhibited an exceptional NH_3_ yield rate of 439.73 µmol cm^−2^ h^−1^ with a FE of 92.4% at −0.6 V vs. RHE in H‐cell. The NH_3_ yield rate was further raised to 655.3 µmol cm^−2^ h^−1^ in the flow cell while maintaining stable electrolysis up to 100 h. Sulfur incorporation plays a dual role in modulating the electronic structure and accelerating interfacial *H transfer in the Cu–Co interface. This induced charge redistribution creates electron‐deficient metal centers and electron‐rich sulfur, which synergistically enhance NO adsorption and activation on Cu sites and facilitate the H_2_O dissociation on Co sites. More importantly, sulfur enables the rapid *H transfer from Co sites to the Cu sites for NO hydrogenation, thereby accelerating NH_3_ production while inhibiting HER. Theoretical calculations and in situ characterizations explicitly elucidate the mechanism of sulfur‐mediated interfacial regulation and investigate the reaction pathway of NO reduction to NH_3_. This work establishes heteroatom‐mediated interface engineering as an effective strategy for optimizing catalytic performance, providing a rational design paradigm for developing advanced NORR electrocatalysts.

## Supporting Information

The detailed experimental section, additional figures tables are listed in the Supporting Information file. The authors have cited additional references within the Supporting Information.

## Conflict of Interests

The authors declare no conflict of interest.

## Supporting information



Supporting Information

## Data Availability

The data that support the findings of this study are available from the corresponding author upon reasonable request.
