# Supplementary material for: Sulfur Mediated Interfacial Proton‐Directed Transfer Boosts Electrocatalytic Nitric Oxide Reduction to Ammonia over Dual‐Site Catalysts
Source: Angew Chem Int Ed Engl. 2025 Jul 9;64(35):e202511398. doi: 10.1002/anie.202511398 (PMC12377428; doi:10.1002/anie.202511398)
Supplement: Supplementary file 1 — Supporting Information [file ANIE-64-e202511398-s001.docx]

***Supporting Information***

**Sulfur Mediated Interfacial Proton-Directed Transfer Boosts Electrocatalytic Nitric Oxide Reduction to Ammonia over Dual-site Catalysts**

Zhenlin Wang,^⊥[a]^ Haiyan Duan,^⊥[a]^ Wenqiang Qu,^[a,c]^ Donglin Han,^[a]^ Xingchi Li,^[a]^ Li Zhu,^[b]^ Xuan Jiang,^[a]^ Danhong Cheng,*^[a]^ Yongjie Shen,^[d]^ Ming Xie,^[e]^ Emiliano Cortes*,^[b]^ Dengsong Zhang*^[a]^

[a] Z. Wang, Assoc. Prof. H. Duan, W. Qu, D. Han, X. Li, X. Jiang, Prof. D. Cheng, Prof. D. Zhang
International Joint Laboratory of Catalytic Chemistry, State Key Laboratory of Advanced Special Steel, Innovation Institute of Carbon Neutrality, Department of Chemistry, College of Sciences, Shanghai University, Shanghai 200444, People’s Republic of China
E-mail: [cdh@shu.edu.cn](mailto:cdh@shu.edu.cn) and [dszhang@shu.edu.cn](mailto:dszhang@shu.edu.cn)

[b] L. Zhu, Prof. E. Cortés
Nanoinstitute Munich, Faculty of Physics, Ludwig-Maximilians-Universität (LMU), Munich 80539, Germany

E-mail: [Emiliano.Cortes@lmu.de](mailto:Emiliano.Cortes@lmu.de)

[c] W. Qu
Department of Chemistry, University of Toronto, 80 St. George Street, Toronto, ON M5S 3H6, Canada

[d] Y. Shen
Institute for Chemical Reaction Design and Discovery (WPI-ICReDD), Hokkaido University, Sapporo 001-0021, Japan

[e] Dr. M. Xie
Department of Chemical Engineering, University of Bath, Bath BA2 7AY, U.K

^⊥^Z. Wang and H. Duan: These authors contributed equally to this work.

**Contents**

1. Materials and Reagents
2. Experimental Section
3. Figures and Tables

Figure S1. Preparation route for the S-Cu@Co/C catalyst.

Figure S2. SEM image of S-Cu@Co MOF precursor.

Figure S3. XRD patterns of the obtained samples by calcining the prepared precursor at different temperatures.

Figure S4. XRD patterns of the catalyst prepared without adding H_3_BTC.

Figure S5. HRTEM image of Cu@Co/C.

Figure S6. HRTEM image of the catalyst prepared without adding H_3_BTC.

Figure S7. Raman spectra of the prepared catalysts.

Figure S8. Isothermal adsorption-desorption curves of S-Cu@Co/C and Cu@Co/C.

Figure S9. Pore size distribution of S-Cu@Co/C and Cu@Co/C.

Figure S10-11. The TEM-EDXS image of S-Cu@Co/C.

Figure S12. S 2p XPS spectra.

Figure S13. EXAFS fitting of Cu K-edge of S-Cu@Co/C.

Figure S14. EXAFS fitting of Co K-edge of S-Cu@Co/C.

Figure S15. Geometrically optimized structure model of S-Co@Cu (a) and S-Cu@Co (b).

Figure S16. CV curves of S-Cu@Co/C and comparison catalyst in PBS electrolyte.

Figure S17. (a) UV-Vis absorption spectra of NH_4_Cl standard solutions for indophenol detection. (b) Corresponding calibration curve used for calculation of NH_3_ concentration.

Figure S18. The effect of different contents of metals and sulfur on NORR.

Figure S19. (a) CA curves for S-Cu@Co/C in NO-saturated PBS electrolyte over 1 h electrolysis at given potentials. (b) UV-Vis absorption spectra of the electrolytes at given potentials stained with indophenol indicator after the NORR over S-Cu@Co/C.

Figure S20. NH_3_ yield rate and FE of the prepared catalysts with and without H_3_BTC at −0.6 V vs. RHE.

Figure S21. CV curves of (a) S-Cu@Co/C and (b) Cu@Co/C under different scan rates from 20 to 120 mV s^-1^.

Figure S22. Nyquist plots of the prepared catalysts.

Figure S23. The NH_3_ yield rate and FE for the NORR over S-Cu@Co/C with PBS or KOH as electrolyte.

Figure S24. Anion chromatographic test of the electrolyte after NORR reaction over S-Cu@Co/C.

Figure S25. Online electrochemical differential mass spectrometry signals of products during the NORR on S-Cu@Co/C.

Figure S26. (a) UV-Vis absorption spectra of N_2_H_4_ standard solutions for Watt and Chrisp detection. (b) Calibration curve used for calculation of N_2_H_4_ concentrations. (c) UV-Vis absorption spectra of NH_2_OH standard solutions. (d) Calibration curve used for calculation of NH_2_OH concentrations.

Figure S27. UV-Vis absorption spectra of N_2_H_4_ (a) and NH_2_OH (b) detected in the electrolyte after 1h of electrolysis over S-Cu@Co/C in H-cell.

Figure S28. In situ electrochemical differential mass spectrometry signals of gas products during the NORR on Cu@Co/C.

Figure S29. FE of NH_3_ and H_2_ over S-Cu@Co/C and Cu@Co/C at -0.6 V vs. RHE in H-cell.

Figure S30. The NORR cycle test on S-Cu@Co/C at -0.6 V vs. RHE.

Figure S31. CA curves for S-Cu@Co/C in NO-saturated PBS electrolyte over 1 h electrolysis for fifteen cycles at -0.6 V vs. RHE.

Figure S32. (a) ^1^H NMR spectra of standard NH_4_Cl solution. (b) Corresponding calibration curve of NMR integral area (NH_4_^+^-N/C_4_H_4_O_4_) against N (NH_4_^+^) concentration.

Figure S33. Comparison of NH_3_ yield and FE between the NMR and colorimetric methods on S-Cu@Co/C.

Figure S34. (a) UV-Vis absorption spectra of N_2_H_4_ and (b) NH_2_OH detected in the electrolyte after 1h of electrolysis over S-Cu@Co/C in flow cell.

Figure S35. The XRD pattern of S-Cu@Co/C after stability test in the flow cell.

Figure S36. The HRTEM images of S-Cu@Co/C after stability test in the flow cell.

Figure S37. The XPS of S-Cu@Co/C after stability test in the flow cell.

Figure S38. Zn-NO battery tests.

Figure S39. NO-TPD spectra of the S-Cu/C and S-Co/C.

Figure S40. The potential-dependent population of interfacial water from in-situ Raman spectra fitting of Cu@Co/C.

Figure S41. Geometrically optimized structural models for water dissociation calculations on Cu and Co sites in Cu@Co/C and S-Cu@Co/C.

Figure S42. Geometrically optimized structural models for water dissociation calculations on S sites in S-Cu@Co/C.

Figure S43. Quasi-in situ electrochemical EPR signals for Cu@Co/C in PBS electrolyte at -0.6 V vs. RHE.

Figure S44. Quasi-in situ electrochemical EPR signals for S-Cu@Co/C in PBS electrolyte at -0.6 V vs. RHE.

Figure S45. The NH_3_ yield rate and FE for the NORR over S-Cu@Co/C with and without TBA as *H scavenging.

Figure S46. The Gibbs free energy diagram for proton transfer from the Co site to the Cu and sulfur site.

Figure S47. PDOS of *NO on S-Cu@Co/C.

Figure S48. Operando ATR-IRAS spectra of electrocatalytic NORR under −0.6 V vs. RHE over Cu@Co/C catalyst.

Figure S49. Online electrochemical differential mass spectrometry signals of products during the NORR on Cu@Co/C.

Figure S50. The diagram of the adsorption of reaction intermediates of NORR over Cu@Co/C after structural optimization.

Table S1. ICP-OES measurement results for S-Cu@Co/C and Cu@Co/C.

Table S2. EXAFS fitting results of the S-Cu@Co/C.

Table S3. Evaluating intrinsic catalytic activity using mass activity and specific activity.

Table S4. Comparison of NH_3_ yield and FE of S-Cu@Co/C with reported NORR electrocatalysts.

Table S5. Comparison of NH_3_ yield and peak power density of S-Cu@Co/C-NO battery with reported metal-NO battery systems.

1. Materials and Reagents

All chemicals were used without further purification. Ultrapure water of 18.2 MΩ was employed for all the experiments. The concentration of the PBS used in the electrochemical tests was 10x (Adamas).

2. Experimental Section

**Synthesis of S-Cu@Co/C and Cu@Co/C.**

For the synthesis of S-Cu@Co/C, 0.75 g 1,3,5-benzenetricarboxylic acid (H_3_BTC), 0.3 g Cu(NO_3_)_2_·3H_2_O, 0.1 g Co(NO_3_)_2_·6H_2_O and 0.3 g 1,2-benzisothiazolin-3-one (BIT) were dissolved in a mixture of 40 ml N, N-dimethylformamide (DMF) and 10 ml methanol. The precursor solution was stirred at room temperature for 30 min, then transferred to a Teflon-lined steel reactor and heated to 120 ºC for 12 h. At the end of the reaction, the obtained powder was washed with methanol and dried at 60 ºC for 12 h in a vacuum oven. The powder was placed in a porcelain boat and heated to 800 ºC (heating rate 5 ºC min^-1^) for 3 h in a stream of N_2_ to yield the S-Cu@Co/C. The Cu@Co/C was synthesized using the same method without adding the BIT.

**Structural characterization.**

X-ray diffraction (XRD, Bruker, D8-Advance, Cu Kα monochromator) was employed to analyze the crystal structure of the catalysts. High-resolution transmission electron microscopy (HR-TEM, JEM-F200) and JEM-ARM 300F Grand ARM for the aberration-corrected high-angle annular dark-field scanning transmission electron microscopy (AC-HAADF-STEM) were utilized to observe the morphological characteristics of the catalysts. The LabRAM HR Evolution (HORIBA) with a 532 nm laser was used to record the Raman spectra. To investigate the composition of the samples, XPS (AXIS Kratos supra+) analysis was performed using Mg-Kα as the excitation source, and the spectrum was calibrated with the C 1s peak at 284.8 eV. The spectrophotometric absorption was obtained using a Cary5000 UV-vis spectrophotometer (Agilent, USA). The Brunauer-Emmett-Teller surface area was determined through nitrogen absorption-desorption isotherm measurements at 77 K, conducted on a nitrogen absorption apparatus (Micromeritics, ASAP 2460). Elemental contents were measured using an ICP-OES (Agilent 5110). The X-ray absorption near edge structure (XANES) and extended X-ray absorption fine structure (EXAFS) spectra were tested at SSRF BL17B1 of the National Facility for Protein Science in Shanghai (NFPS), Shanghai Advanced Research Institute. The EXAFS fitting was performed and analyzed with the Athena and Artemis programs of the Demeter data analysis packages that utilized the FEFF6 program to fit the EXAFS data.

**Electrochemical measurements.**

**H-cell measurement:** For electrochemical measurements, an H-type electrochemical cell was utilized, which was sealed and separated by a Nafion 117 membrane, along with a CHI working station (CHI 660E, Chenghua Inc., Shanghai, China). Before use, the Nafion membrane was pre-treated at 80 °C for 1 h in H_2_O_2_ (5 wt%) aqueous solution. Then, it was in 0.5 M H_2_SO_4_ for 2 h and finally in water for 6 h at room temperature. The tests employed a three-electrode system, the catalyst coated in carbon paper (1 cm^2^, Toray TGP-H-060) as the working electrode, an Ag/AgCl electrode serving as the reference electrode, and a graphite rod as the counter electrode. All potentials presented in this study were referenced to a reversible hydrogen electrode (RHE) according to the Nernst equation: E (V *vs.* RHE) = E (V *vs.* Ag/AgCl) + 0.198 V + 0.059 × pH. Electrolysis experiments were performed in 0.1 M PBS electrolyte. Before the electrolysis measurements, the cathodic compartment was purged with high-purity Ar gas for 30 min to eliminate residual N_2_ and O_2_. Subsequently, a mixed gas (10% NO) was introduced and maintained constant during the tests. The catalyst ink was prepared by ultrasonically dispersing 5 mg of catalyst in 300 μL ethanol, 170 μL ultrapure water, and 30 μL Nafion (5 wt.%, Du Pont). The working electrode was prepared by depositing 100 μL of the catalyst ink onto the carbon paper. LSV curves were obtained at a rate of 5 mV s^-1^ before the electroreduction test, without IR compensation. Nyquist plots were measured across frequencies ranging from 10^6^ Hz to 10^-2^ Hz. After 1 hour of electrolysis, the aqueous products in the cathodic compartment were detected using the colorimetric method. For the recycling durability tests, a fresh electrolyte was used for each cycle.

**Flow cell measurement:** A commercial flow cell electrolyzer (101017, Gaoss Union Technology Co., LTD) was used for flow cell tests. The working electrode was fabricated by depositing the catalyst ink onto a gas diffusion electrode (GDE, YLS-30T, 1 cm^2^). The catalyst-coated side faced the cathode compartment, while the other side was in contact with the gas chamber. The cathode and anode compartments were separated by a proton exchange membrane (PEM, Nafion 117). A platinum foil served as the anode, and Ag/AgCl acted as the reference electrode. The gas chamber was purged with high-purity Ar gas at a flow rate of 15 mL min^-1^ for 30 min and then NO was introduced at the same flow rate and maintained constant during the electrochemical tests. The electrolyte was continuously circulated using a pump. The aqueous products in the cathodic compartment were detected using both the colorimetric method and ^1^H nuclear magnetic resonance (^1^H NMR).

**Zn-NO battery test:** A carbon paper (1 cm^2^) loaded with S-Cu@Co/C served as the cathode, operating in PBS electrolyte. Meanwhile, a polished high-purity Zn plate (1 cm^2^) was employed as the anode in a 1 M KOH electrolyte. The two compartments were separated using a Nafion 117 membrane. During the discharge process, the Zn-NO electrochemistry indicated that electrochemical NO reduction was driven by the dissolution of the Zn plate.

**Determination of products.**

**Determination of ammonia**: The yield of NH_3_ during the electrolysis process was quantified through spectrophotometric measurements employing the indophenol blue method and ^1^H nuclear magnetic resonance. A specific volume of cathodic electrolyte was collected after 1 hour of electrolysis and subsequently diluted to fall within the detection range. This diluted electrolyte (2 mL) was then combined with 2 mL of Reagent A, 1 mL of Reagent B, and 200 μL of Reagent C. Specifically, Reagent A consisted of a 1 M NaOH solution containing 5 wt% salicylic acid and 5 wt% trisodium citrate dihydrate. Reagent B is a 0.05 M sodium hypochlorite solution, while Reagent C is a 1 wt% sodium nitroprusside solution. The resulting mixture was incubated for 1 hour at room temperature, following which the absorption at 655 nm was measured using a UV-vis spectrophotometer. A series of standard NH_4_Cl solutions with varying concentrations were used to create a calibration plot for NH_3_ quantification. The NH_3_ yield rate and Faradaic efficiency (FE) were determined using specific equations:

NH_3_ yield rate (μmol h^-1^ cm^-2^) =$\frac{C_{{NH}_{3}}\times V}{17\times t\times A}$ (1)

NH_3_ Faradaic efficiency (%) =$\frac{{5\times F\times C}_{{NH}_{3}}\times V}{17\times Q\times{10}^{6}}$×100 % (2)

the *C*_NH3_ (μg mL^-1^) is the NH_3_ concentration, *V* (mL) is the volume of the electrolyte, t (h) is the reaction time and A (cm^2^) is the surface area of carbon paper (1 cm^2^). F (96485 C mol^-1^) is the Faraday constant and Q (C) is the total quantity of applied electricity.

To obtain the standard calibration plot for NH_3_ quantification using nuclear magnetic resonance (^1^H NMR, 600 MHz), NH_4_Cl of a known concentration was dissolved. This involved mixing 40 μL of the NH_4_Cl solution with 20 μL of 0.5 M HCl, 20 μL of 3.6 mM maleic acid (serving as an internal standard), and 500 μL of D_2_O. The NH_3_ quantification was achieved by integrating the triplet relative to the standard maleic acid peak at 6.23 δ. For testing the NH_3_ yield in the electrolyte after NO electroreduction, the electrolyte was initially diluted to a specific concentration, and the same method was employed.

**Determination of hydrazine hydrate (N_2_H_4_):** The concentration of N_2_H_4_ in the electrolyte was determined quantitatively using the Watt and Chrisp method. This involved preparing a color reagent by combining 300 mL of ethanol, 5.99 g of para-(dimethylamino) benzaldehyde, and 30 mL of concentrated HCl. Subsequently, 4 mL of electrolyte was mixed with 1 mL of 1 M KOH, followed by the addition of 5 mL of the color reagent. After allowing the mixture to incubate at room temperature for 20 minutes, the absorbance of the solution was measured at a wavelength of 455 nm using a UV-vis spectrophotometer. A standard calibration curve for N_2_H_4_ quantification was obtained by using a series of standard concentrations of N_2_H_4_ solution.

**Determination of Hydroxylamine (NH_2_OH):** 1 mL electrolyte was taken and diluted with 1 mL 0.05 M phosphate buffer solution and 800 μL ultrapure water. Then, 1 mL of 1% 8-quinolinol (w/v ethanol), 200 μL of 1 M trichloroacetic acid and 1 mL of 1 M Na_2_CO_3_ solution were added into the above solution. The solution was then heated for 1 min at 100 ºC, and then its absorbance at 707 nm was measured by a UV-visible absorption spectrophotometer when cooled to room temperature. The calibration curves for different concentrations of hydroxylamine were obtained by recording UV-visible spectra.

**In-situ Raman measurement.**

A commercial Raman cell electrolyzer with a gas chamber, cathode, and anode compartments for the in-situ Raman tests. The tests were conducted on LabRAM HR Evolution (HORIBA) using a 532 nm laser. The electrolyte was continuously cycled through the two compartments at 25 mL min^-1^ under the pump drive and the NO with 15 mL min^-1^ for the tests. The working electrode was prepared by dropping the catalyst ink loaded on the gas diffusion electrode (3 × 3 cm^2^). The Ag/AgCl is the reference electrode and the graphite rod is the counter electrode.

**NO-TPD measurement.**

The NO temperature programmed desorption (NO-TPD) tests were conducted on a VDsorb-91i chemisorption apparatus. 20 mg of catalyst was placed in a glass tube, and was pre-treated by He (30 mL min^-1^) flow at 200 ºC for 30 min and then cooled down to 50 ºC. The adsorption of NO was carried out at 50 ºC (10 %). After purging with He for 30 min to remove the physical adsorption of NO, the sample underwent heating at a rate of 10 ºC min^-1^, increasing the temperature from 50 ºC to 500 ºC. The MS signals of m/z = 30 were collected by the MS detector (OMNISTAR).

**Online differential electrochemical mass spectrometry (DEMS) measurement.**

The QAS 100 spectrometer (Ling Lu Instruments, Shanghai) was employed for the online differential electrochemical mass spectrometry (DEMS) measurements. During the test, the vacuum system keeps the vacuum degree below 1×10^-7^ Pa. The three-electrode system was used for the measurements, and the catalyst was dropped on the carbon cloth (1 cm^2^, Toray SCC130) as the working electrode, Ag/AgCl (saturated KCl electrolyte, 3.5 M) and the platinum wire as the reference electrode and counter electrode, respectively. The electrolyte was pre-purged with high-purity Ar gas for 30 min to remove the residual air. Then, the NO was fed during the tests. The experiments were employed by LSV tests and the potential was increased from open circuit potential (OCP) to -0.6 V vs*.* RHE with a 10 mV s^-1^ scat rate.

**In situ Operando attenuated total reflection infrared absorption spectroscopy.**

In situ electrochemical operando ATR-IRAS measurements were conducted using a Bruker INVENIO R spectrometer, which featured a liquid nitrogen-cooled mercury cadmium telluride A (MCT-A) detector. The spectroelectrochemical cell was custom-designed by Ling Lu Instruments, Shanghai. The working electrode was fabricated by depositing the catalyst ink onto a face-angled Si crystal. For the test, an Ag/AgCl electrode served as the reference electrode, while a Pt wire was the counter electrode. The electrolyte was purged with Ar for 30 min to remove the residual oxygen. The background was recorded at open circuit potential (OCP). Then, the NO was injected and the potential was settled at -0.6 V vs. RHE and maintained for 30 min. The NO adsorption was measured without applying bias potential for 30 min. The spectra in the measurements were shown by the absorbance. A negative peak indicates the consumption of the substance or intermediate, whereas a positive peak signifies its production or increase.

**Quasi-in situ EPR measurements.**

Quasi-in situ EPR was performed on the EPR 200-Plus instrument. For the test, the electrochemical reaction was carried out in H-cell with catalysts coated on carbon paper. For each sample, the electrolyte was purged with Ar for 10 min and then baseline was tested before starting electrolysis. Then the electrolyte was taken after different electrolysis times and tested. 5,5-dimethyl-1-pyrroline-N-oxide (DMPO) was used as a trapping agent and was added at 5 mM for each tests.

**DFT calculations.**

The DFT as implemented in the Vienna Ab initio simulation package (VASP) in all calculations^[1]^. The exchange-correlation potential is described by using the generalized gradient approximation of Perdew-Burke-Ernzerhof (GGA-PBE)^[2]^. The projector augmented-wave (PAW) method was employed to treat interactions between ion cores and valence electrons^[3]^. The plane-wave cutoff energy was fixed to 450 eV. Given structural models were relaxed until the Hellmann-Feynman forces smaller than -0.02 eV Å^-1^ and the change in energy smaller than 10^-5^ eV was attained. The Grimme’s DFT-D3 methodology was used to describe the dispersion interactions among all the atoms in adsorption models^[4]^. The Gamma-centered k-points samplings were set to 1 × 2 × 1 for the model. The vacuum space along the z-direction was set to be 15 Å. Spin-polarized calculations were performed for this calculation.

The adsorption energy (E_ads_) is calculated using the equation:

E_ads_ = E_(total)_ - E_(slab)_ - E_(NO)_

E_(total)_ is the total energy of an optimized slab with the NO adsorbed, E_(slab)_ is the energy of a relaxed clean slab, and E_(NO)_ is the energy of a NO molecule.

The Gibbs free energy change is defined as:

ΔG = ΔE + ∆E_ZPE_ – TΔS

where ΔE is the reaction energy of reactant and product molecules adsorbed on catalyst surface, obtained from DFT calculations, ΔZPE and ΔS are the zero-point energy difference and the entropy change between the products and reactants, respectively, and T is the temperature (298.15 K).

3. Figures and Tables


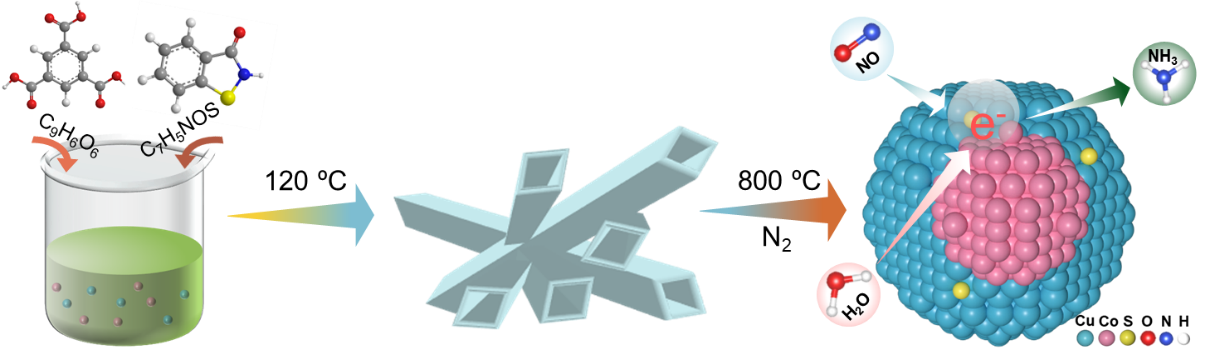


Figure S1. Preparation route for the S-Cu@Co/C catalyst.


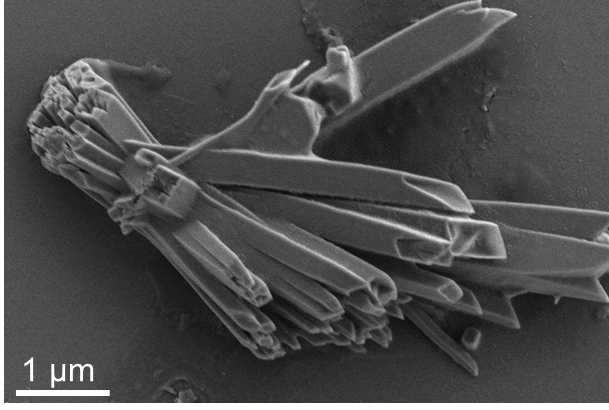


Figure S2. SEM image of S-Cu@Co MOF precursor.


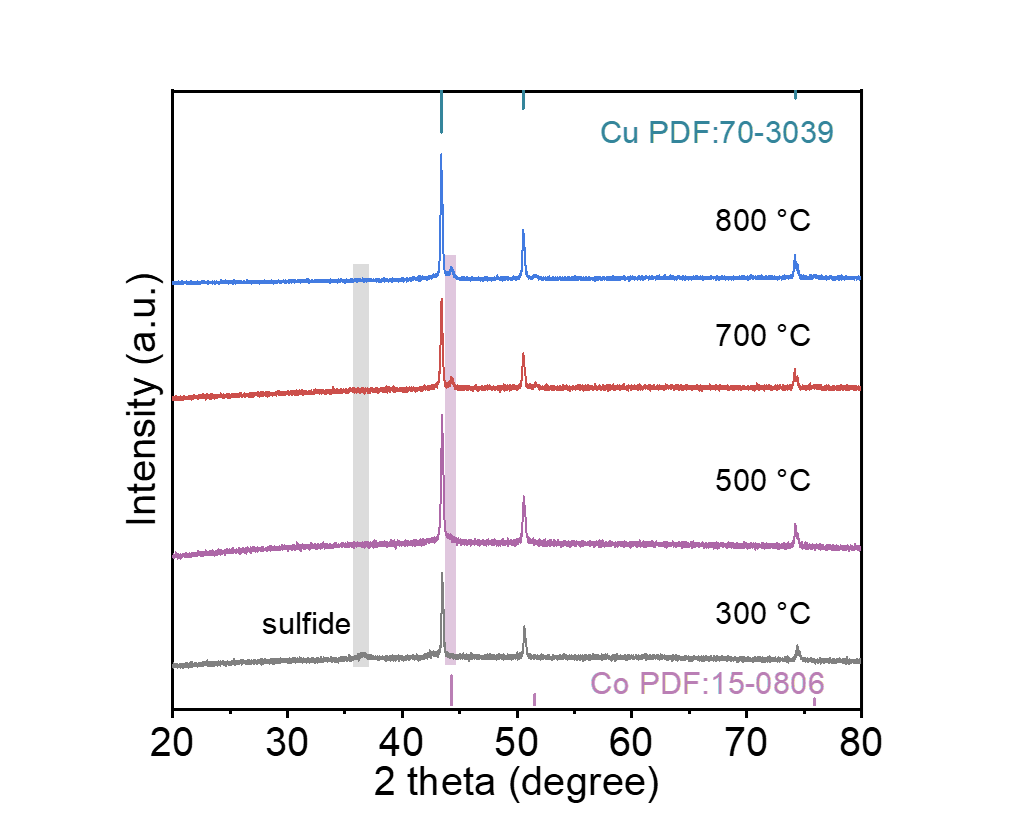


Figure S3. XRD patterns of the obtained samples by calcining the prepared precursor at different temperatures.


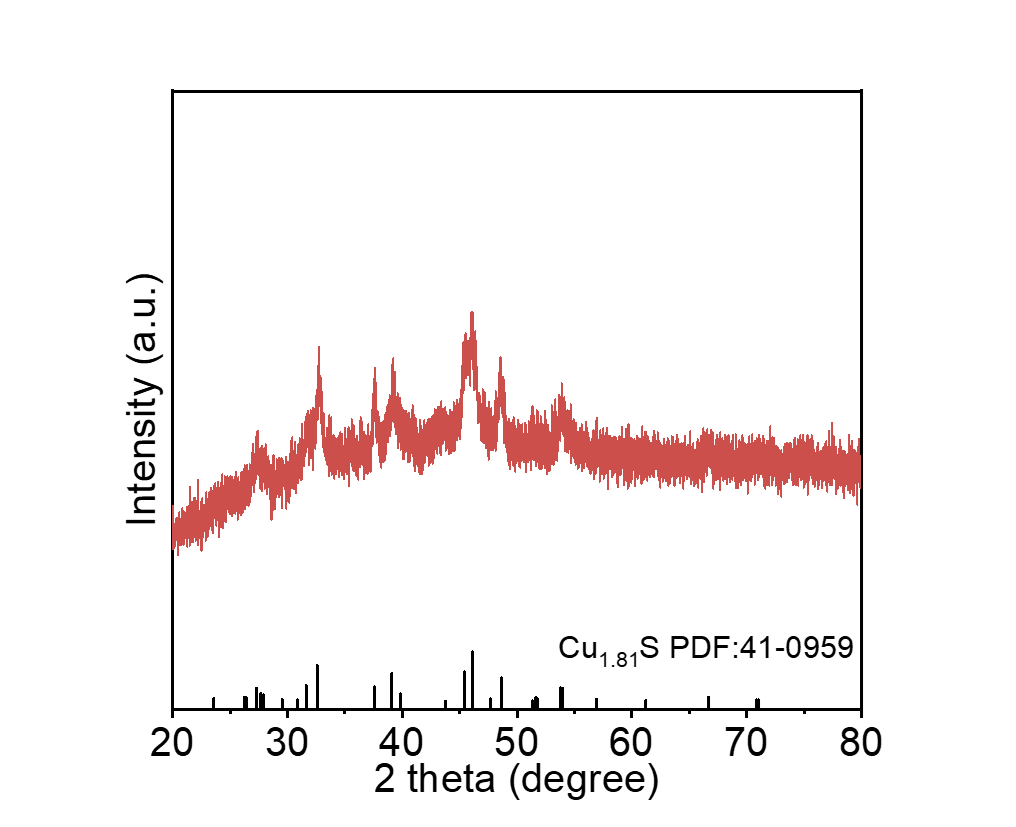


Figure S4. XRD patterns of the catalyst prepared without adding H_3_BTC.


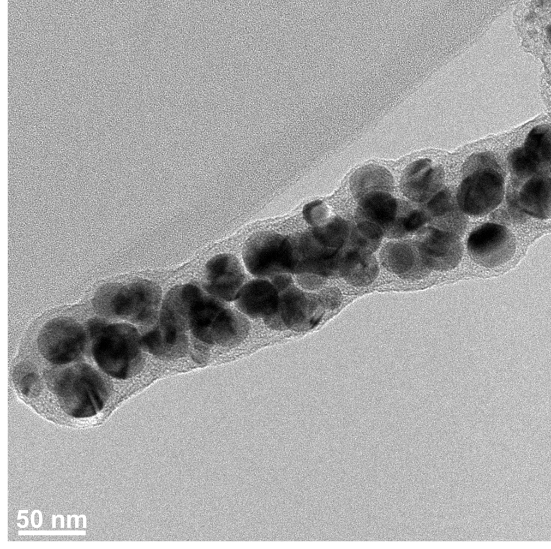


Figure S5. HRTEM image of Cu@Co/C.


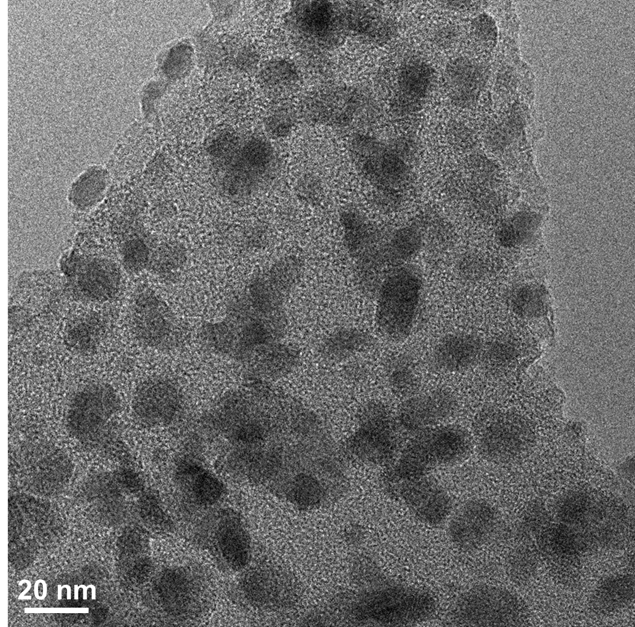


Figure S6. HRTEM image of the catalyst prepared without adding H_3_BTC.


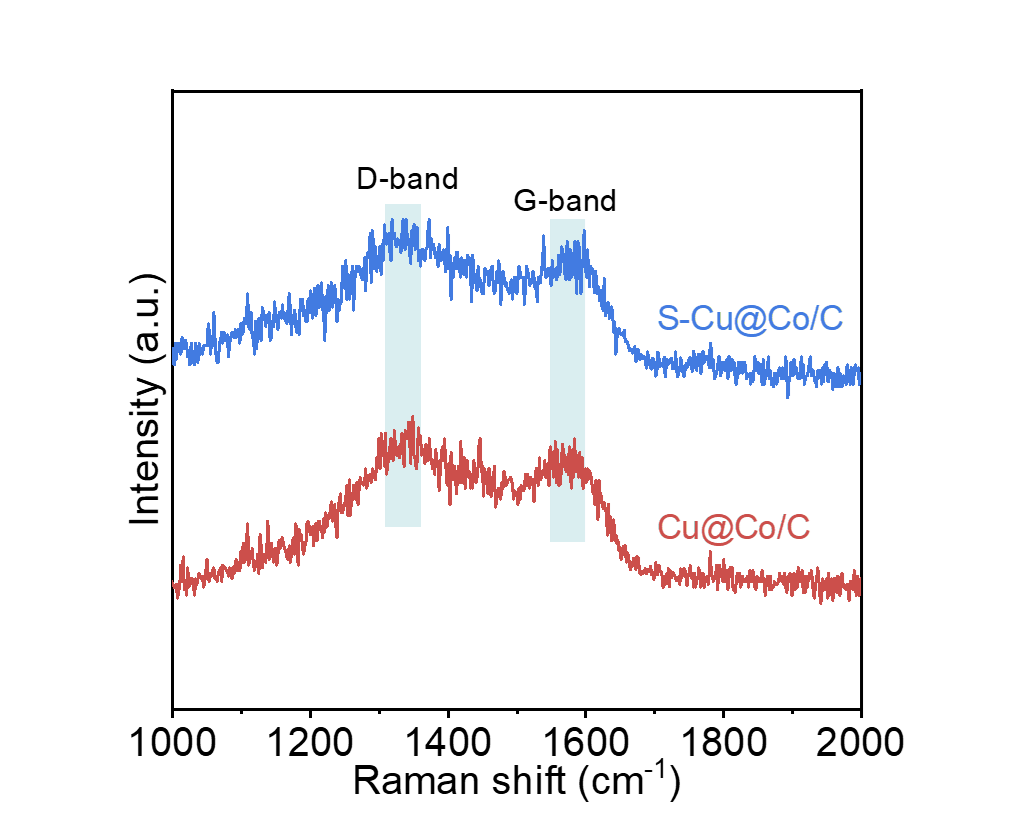


Figure S7. Raman spectra of the prepared catalysts.


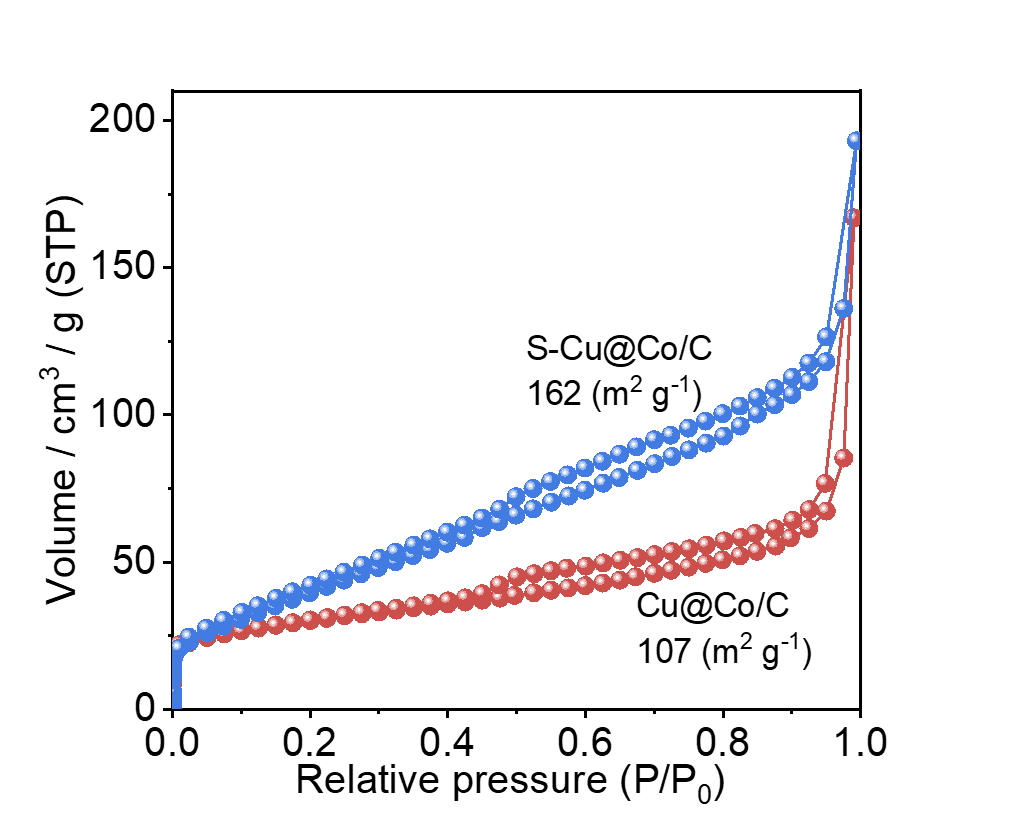


Figure S8. Isothermal adsorption-desorption curves of S-Cu@Co/C and Cu@Co/C.


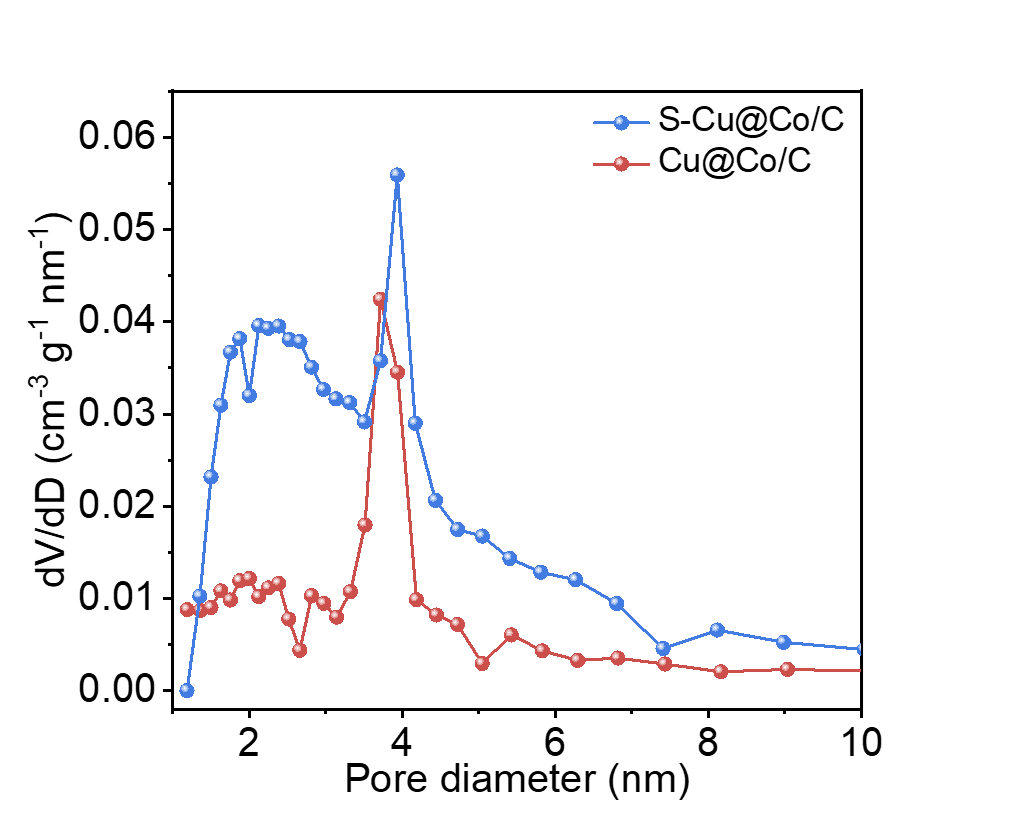


Figure S9. Pore size distribution of S-Cu@Co/C and Cu@Co/C.

The pore size distribution of S-Cu@Co/C ranged from 1.5 nm to 7 nm, while the pore size of Cu@Co/C was concentrated at 3.9 nm.


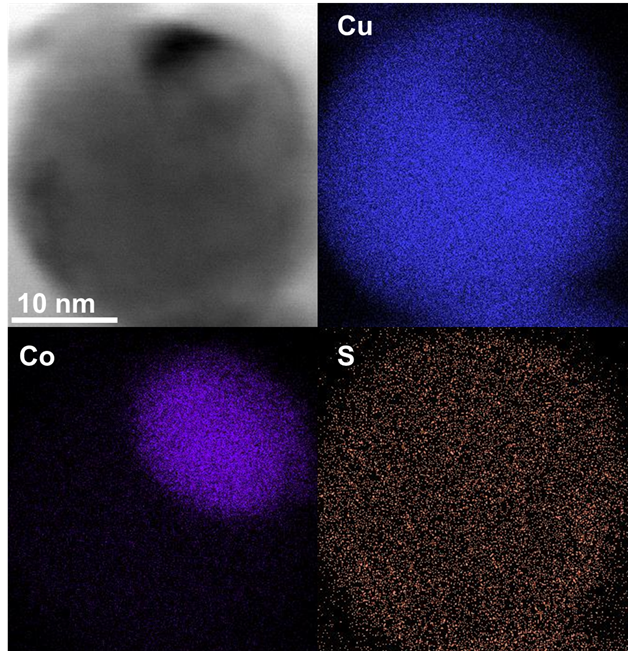


Figure S10. The TEM-EDS image of S-Cu@Co/C.


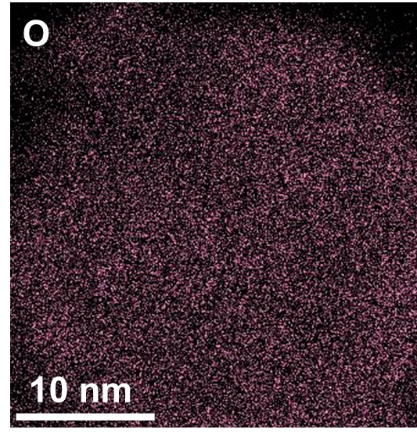


Figure S11. The TEM-EDS image of S-Cu@Co/C.


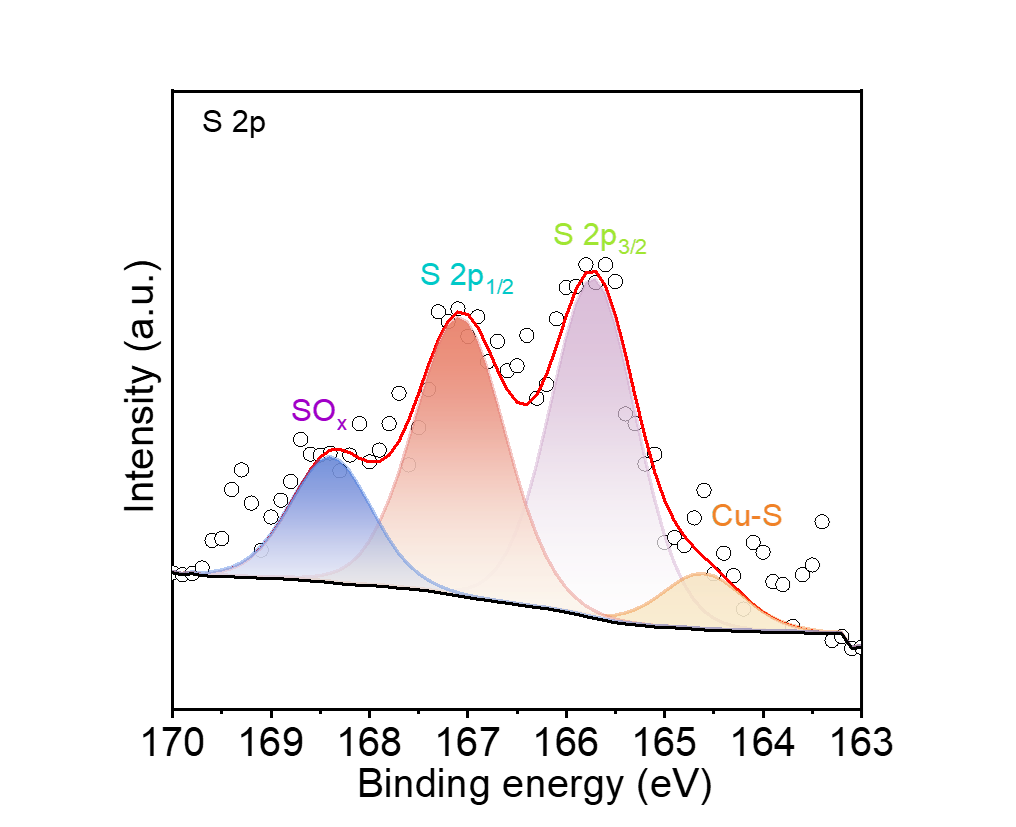


Figure S12. S 2p XPS spectra.


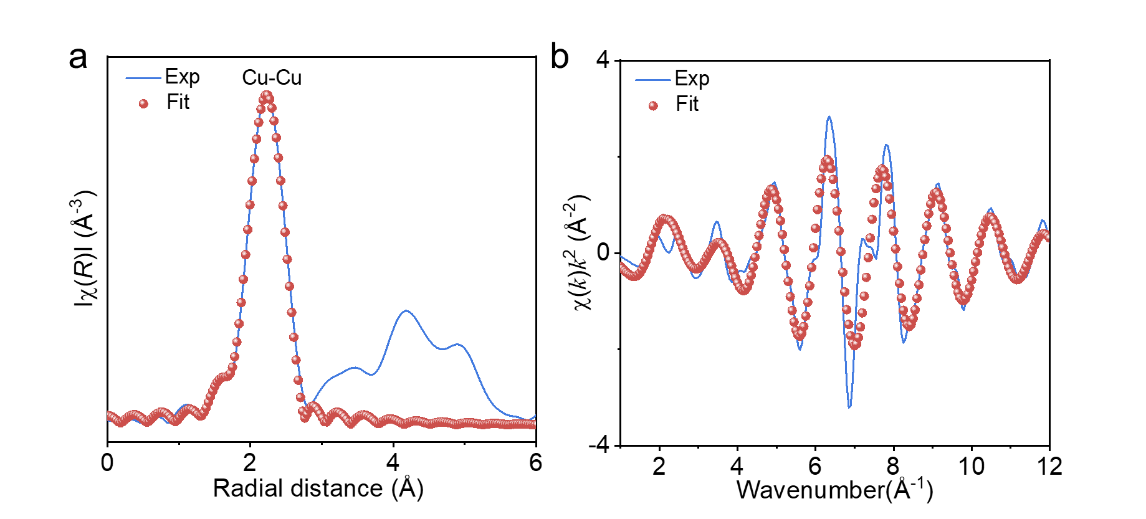


Figure S13. EXAFS fitting of Cu K-edge of S-Cu@Co/C.


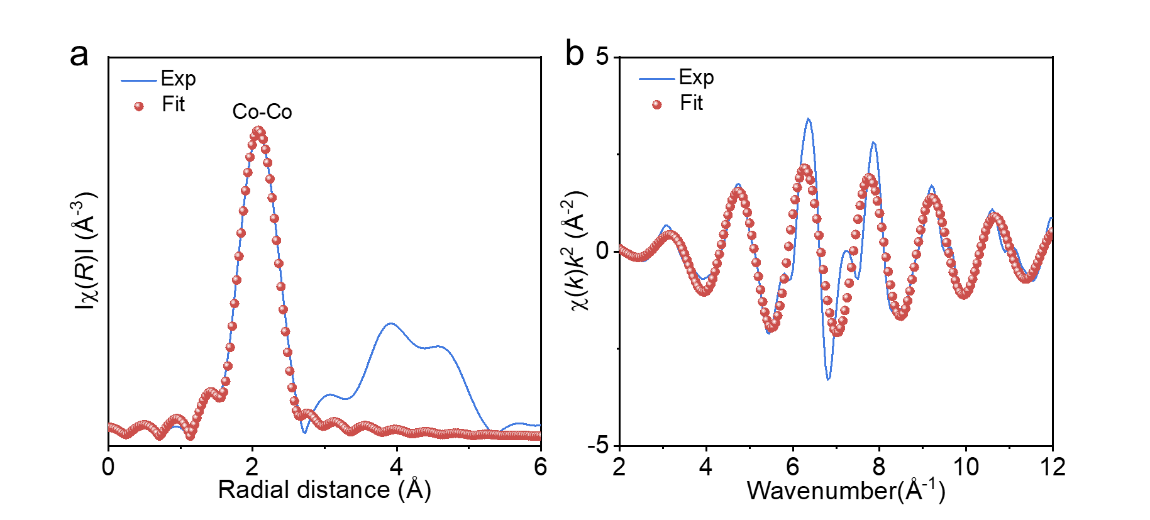


Figure S14. EXAFS fitting of Co K-edge of S-Cu@Co/C.


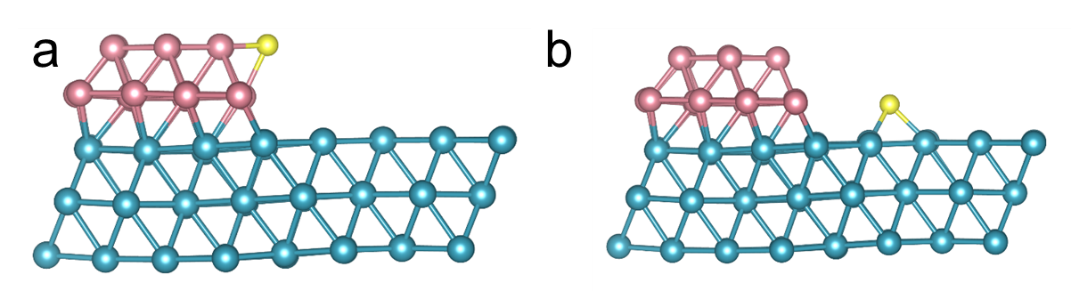


Figure S15. Geometrically optimized structure model of S-Co@Cu (a) and S-Cu@Co (b).

The DFT calculations confirmed that the S on the Cu site (-4.67 eV) is more stable than on the Co site (-4.40 eV).


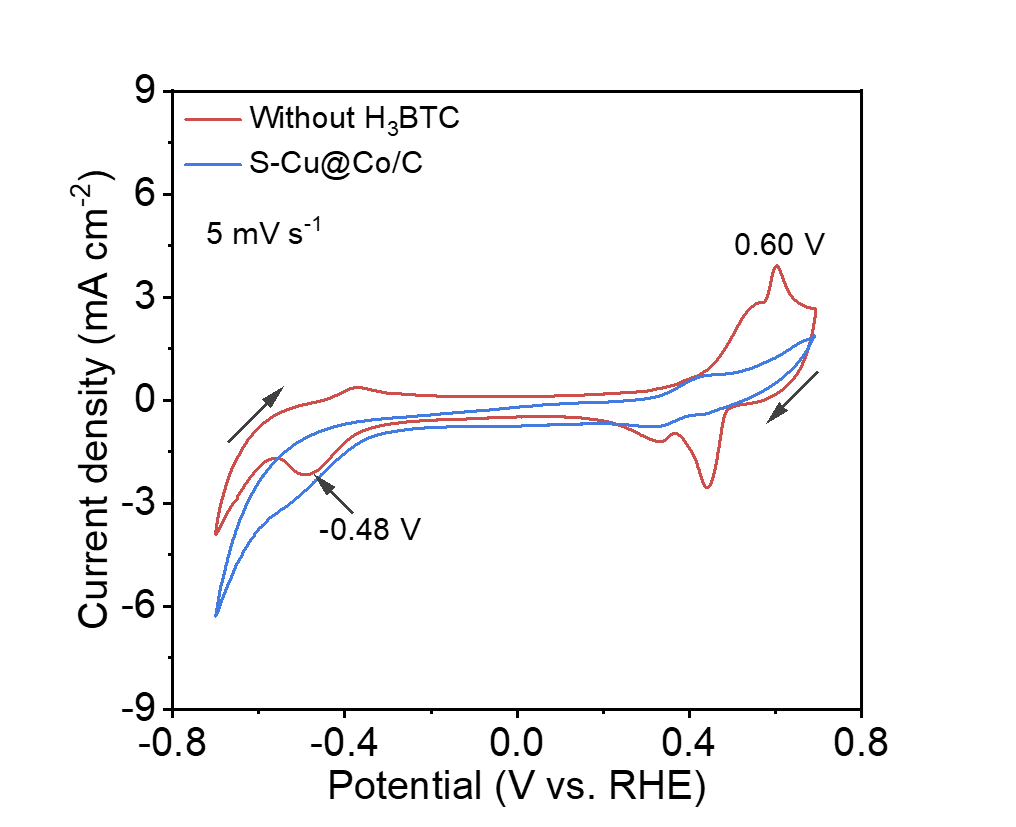


Figure S16. CV curves of S-Cu@Co/C and comparison catalyst in PBS electrolyte.


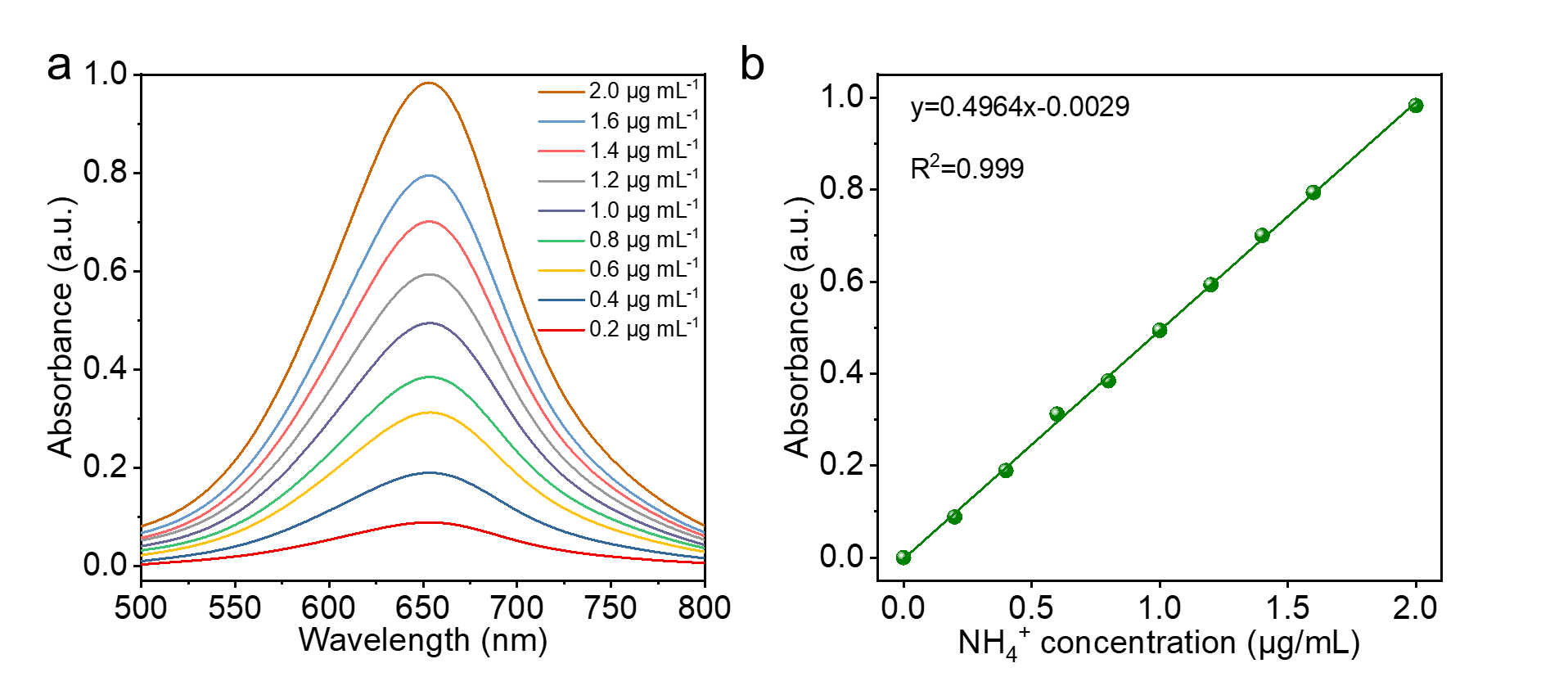


Figure S17. (a) UV-Vis absorption spectra of NH_4_Cl standard solutions for indophenol detection. (b) Corresponding calibration curve used for calculation of NH_3_ concentration.


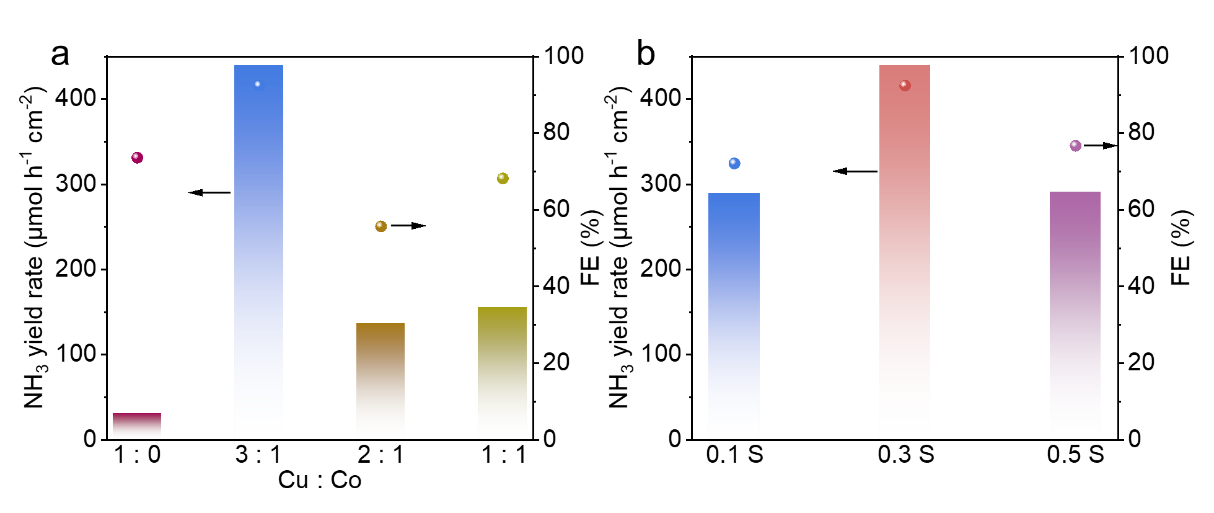


Figure S18. The effect of different contents of metals and sulfur on NORR.

The effects of different metals and sulfur content on NORR were first investigated. The results suggested that the ratio of Cu to Co was 3:1, and the sulfur addition of 0.3 presented the most outstanding NORR performance.


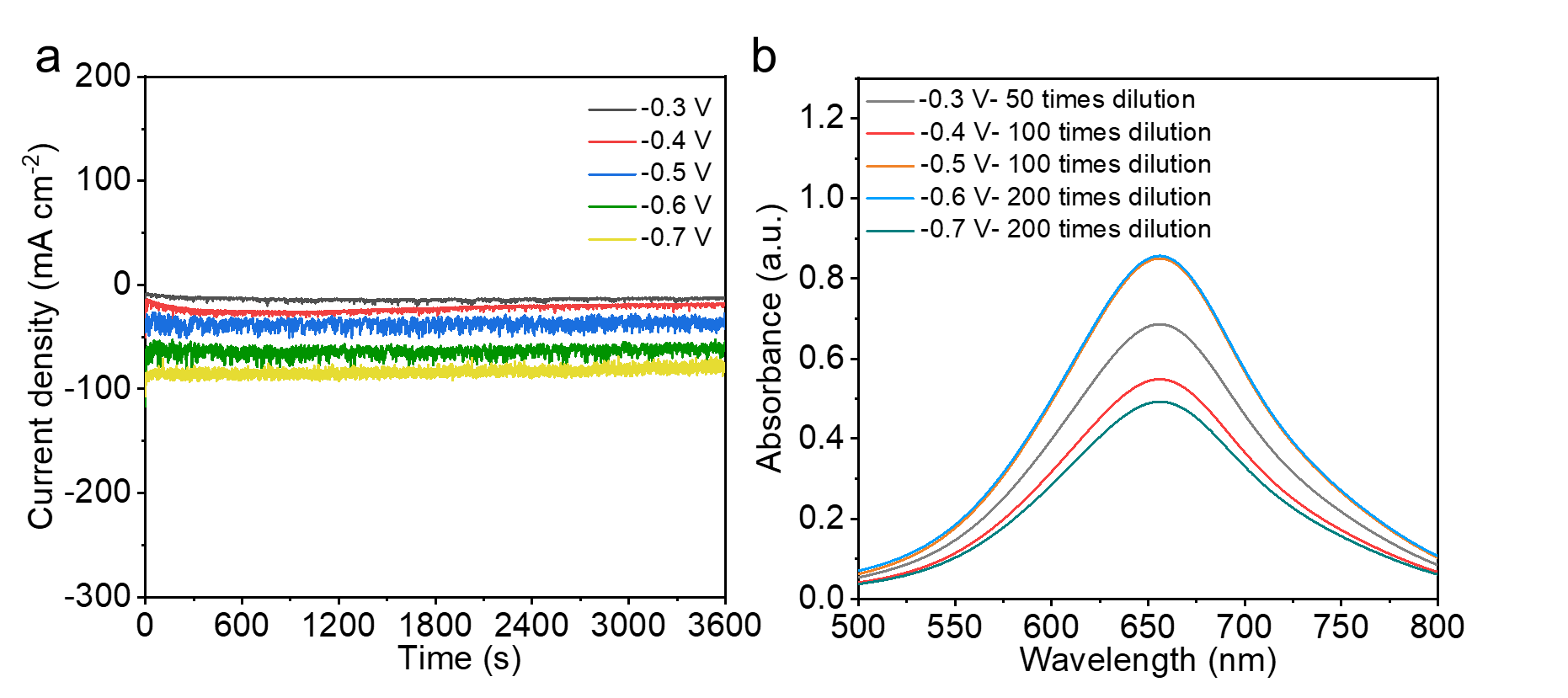


Figure S19. (a) CA curves for S-Cu@Co/C in NO-saturated PBS electrolyte over 1 h electrolysis at given potentials. (b) UV-Vis absorption spectra of the electrolytes at given potentials stained with indophenol indicator after the NORR over S-Cu@Co/C.

Figure S20. NH_3_ yield rate and FE of the prepared catalysts with and without H_3_BTC at −0.6 V vs. RHE.


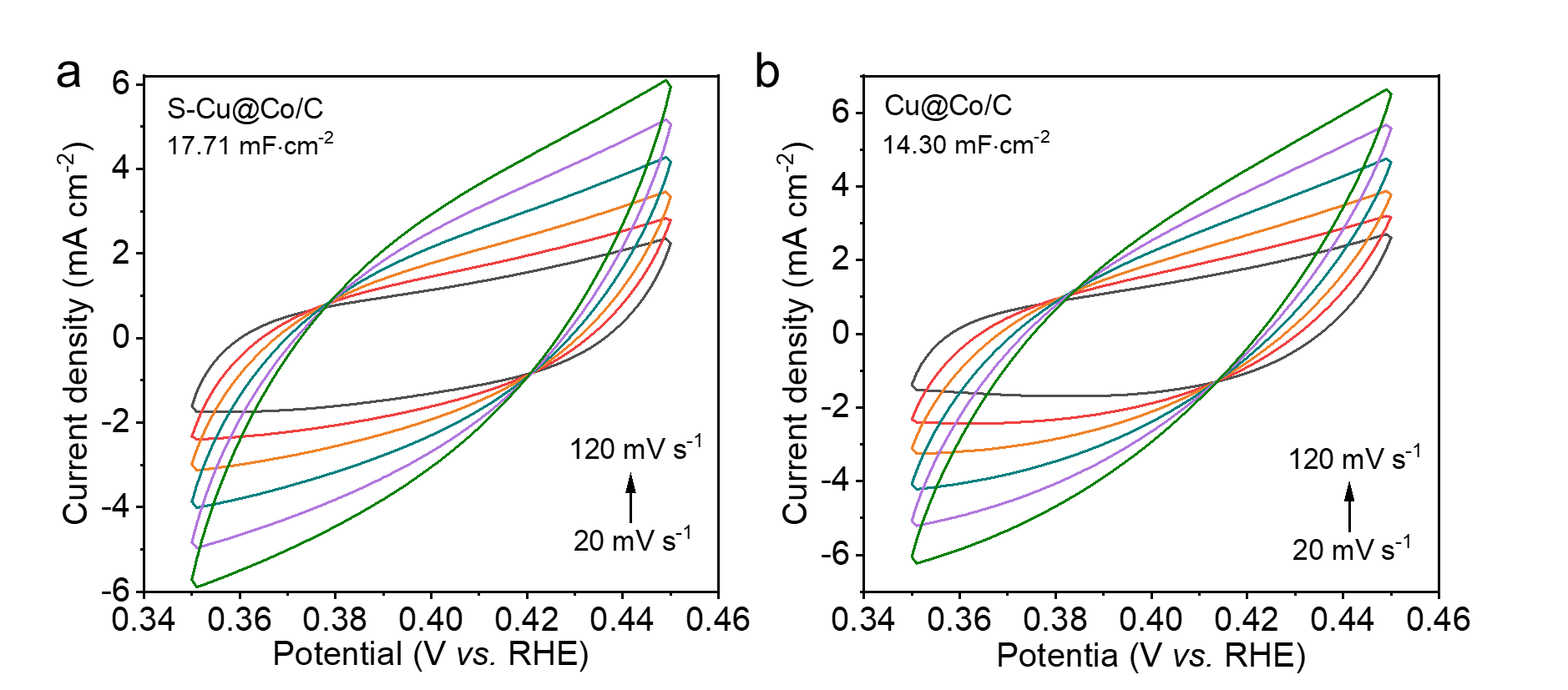


Figure S21. CV curves of (a) S-Cu@Co/C and (b) Cu@Co/C under different scan rates from 20 to 120 mV s^-1^.

The CV curves at different scan rates were used to calculate the catalyst's double-layer capacitance (*C*_dl_). It can be found that the S-Cu@Co/C has a larger C_dI_ value.


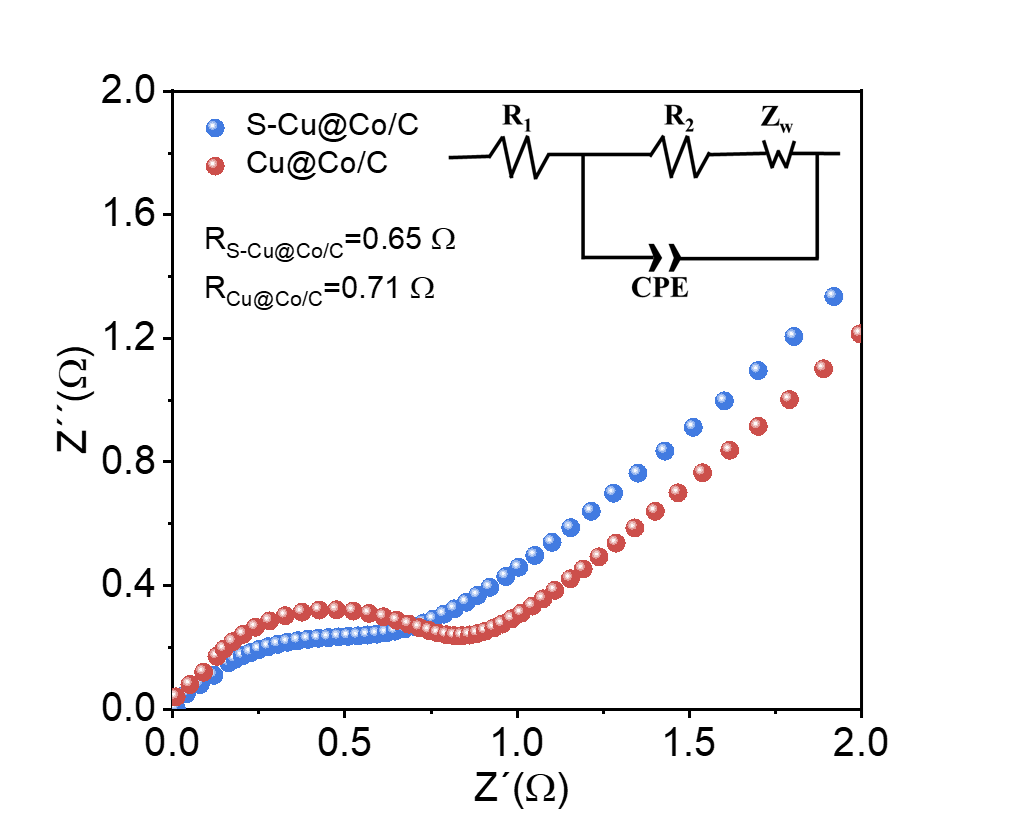


Figure S22. Nyquist plots of the prepared catalysts.

Electrochemical impedance shows that the S-Cu@Co/C has the smallest charge transfer resistance (R_ct_), indicating faster electron transfer in the electrocatalytic NORR process.


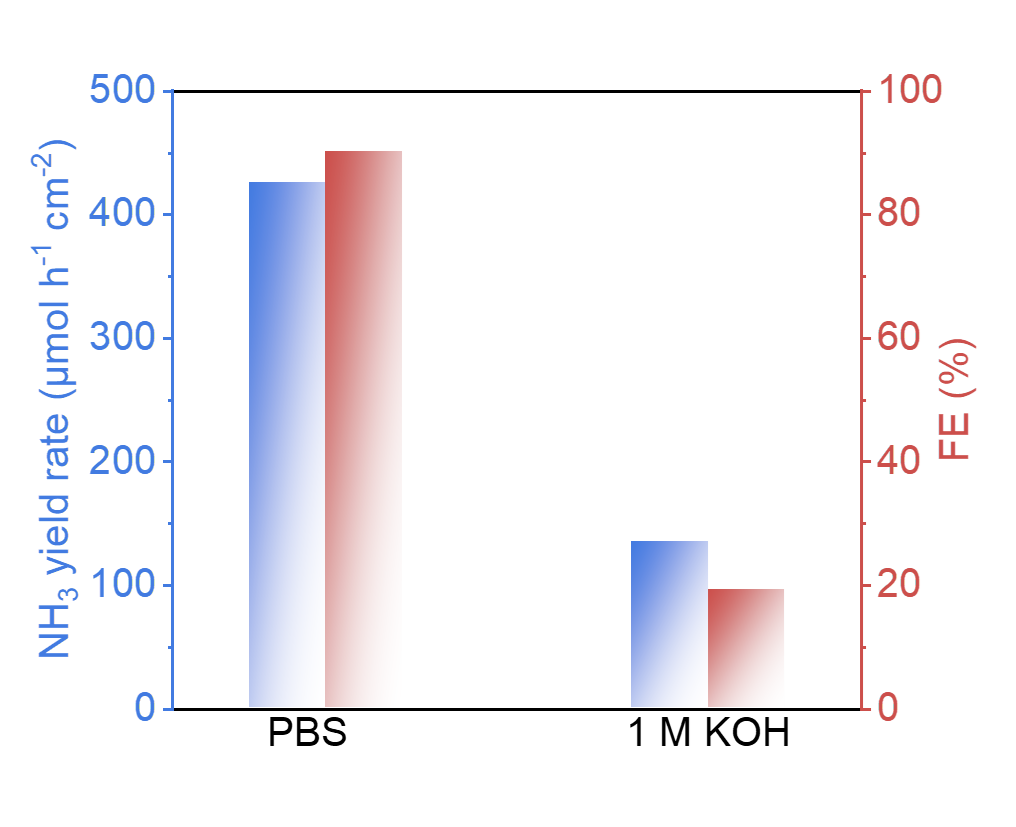


Figure S23. The NH_3_ yield rate and FE for the NORR over S-Cu@Co/C with PBS or KOH as electrolyte.


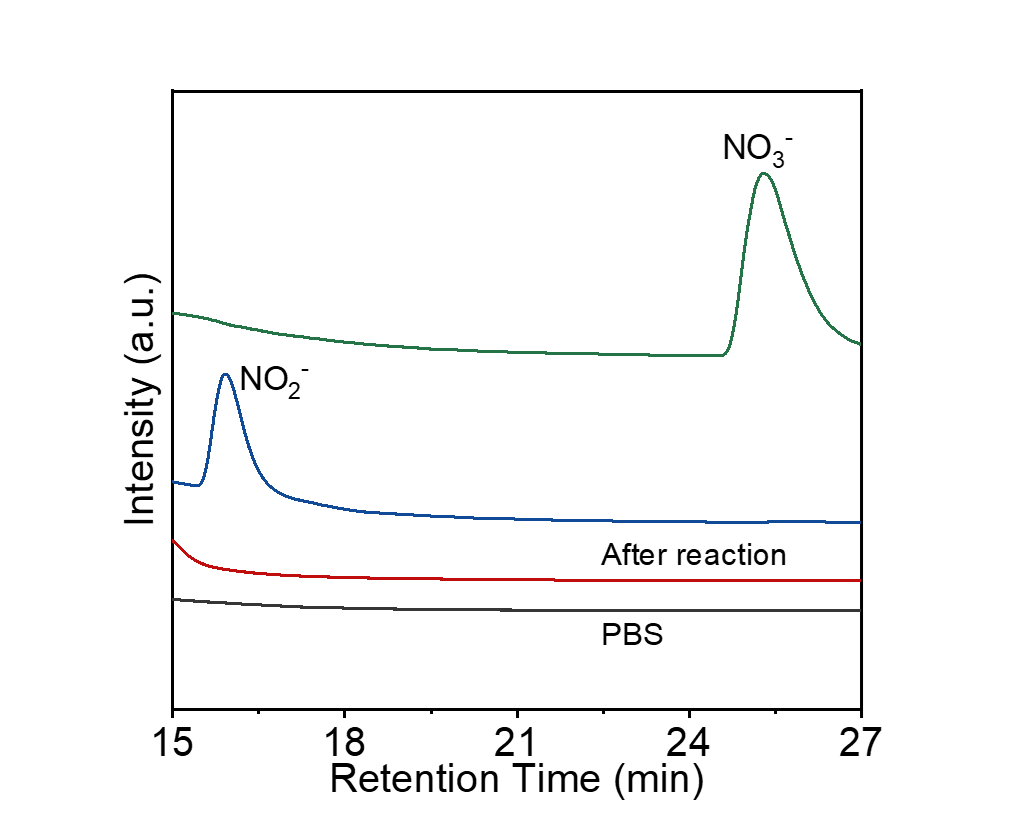


Figure S24. Anion chromatographic test of the electrolyte after NORR reaction over S-Cu@Co/C. The fresh PBS, NO_3_^-^ and NO_2_^-^ solutions were used as controls.

Anion chromatography was employed to detect if NO_3_^-^ or NO_2_^-^ were generated during the NORR on S-Cu@Co/C. The standard NO_3_^-^ and NO_2_^-^ shows that no NO_3_^-^ and NO_2_^-^ was produced in the reaction.


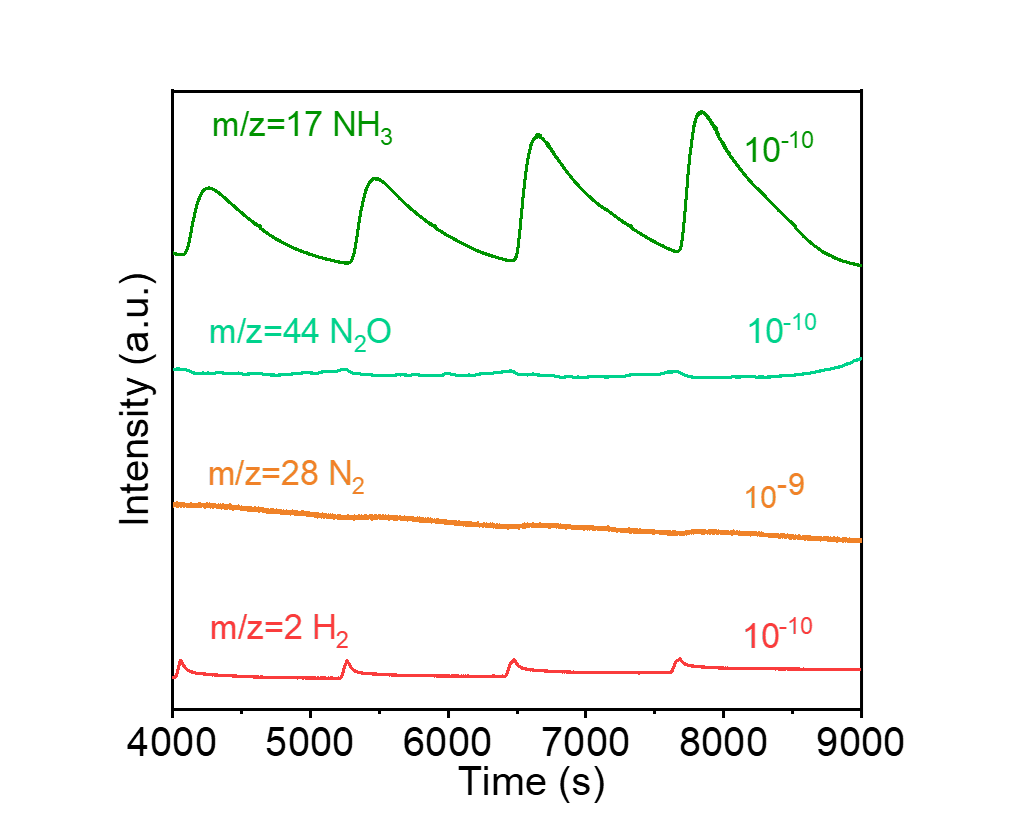


Figure S25. Online electrochemical differential mass spectrometry signals of products during the NORR on S-Cu@Co/C.

The DEMS results show that H_2_ is the main gas byproduct, with negligible production of N_2_O and N_2_ during the NORR process.


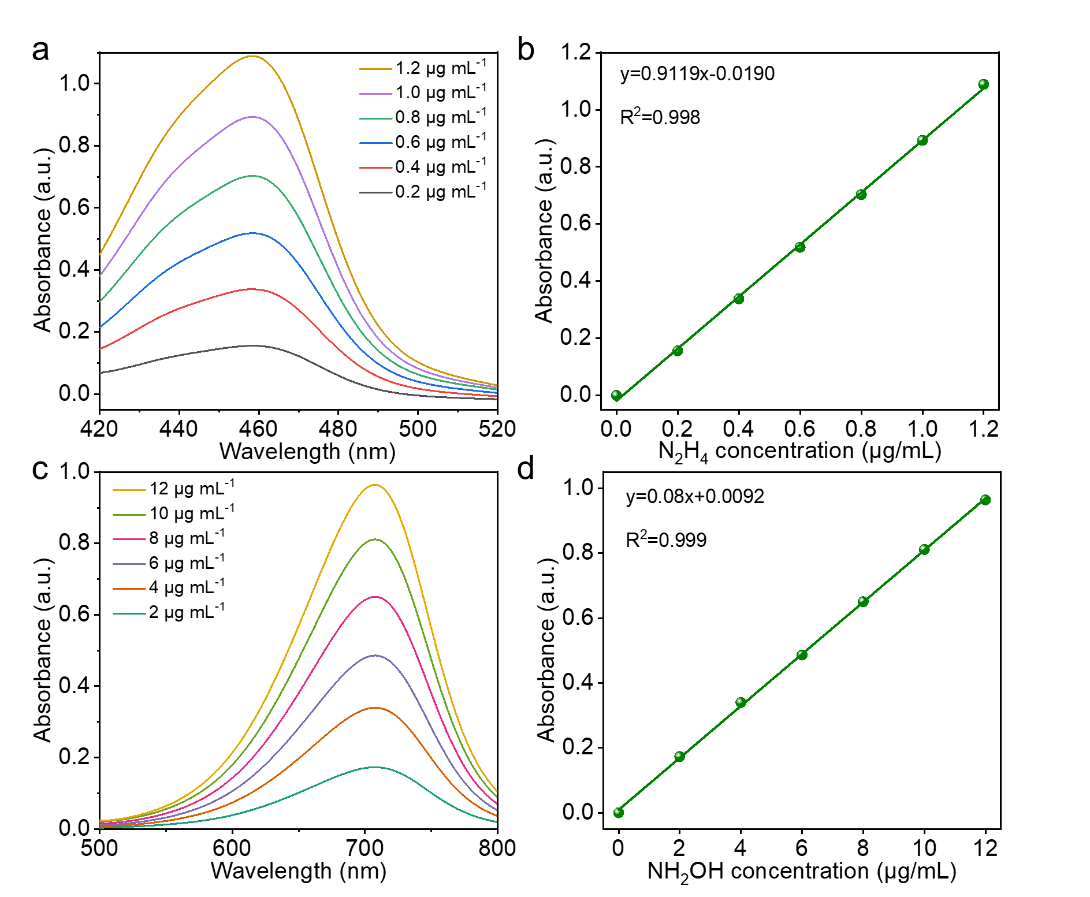


Figure S26. (a) UV-Vis absorption spectra of N_2_H_4_ standard solutions for Watt and Chrisp detection. (b) Calibration curve used for calculation of N_2_H_4_ concentrations. (c) UV-Vis absorption spectra of NH_2_OH standard solutions. (d) Calibration curve used for calculation of NH_2_OH concentrations.


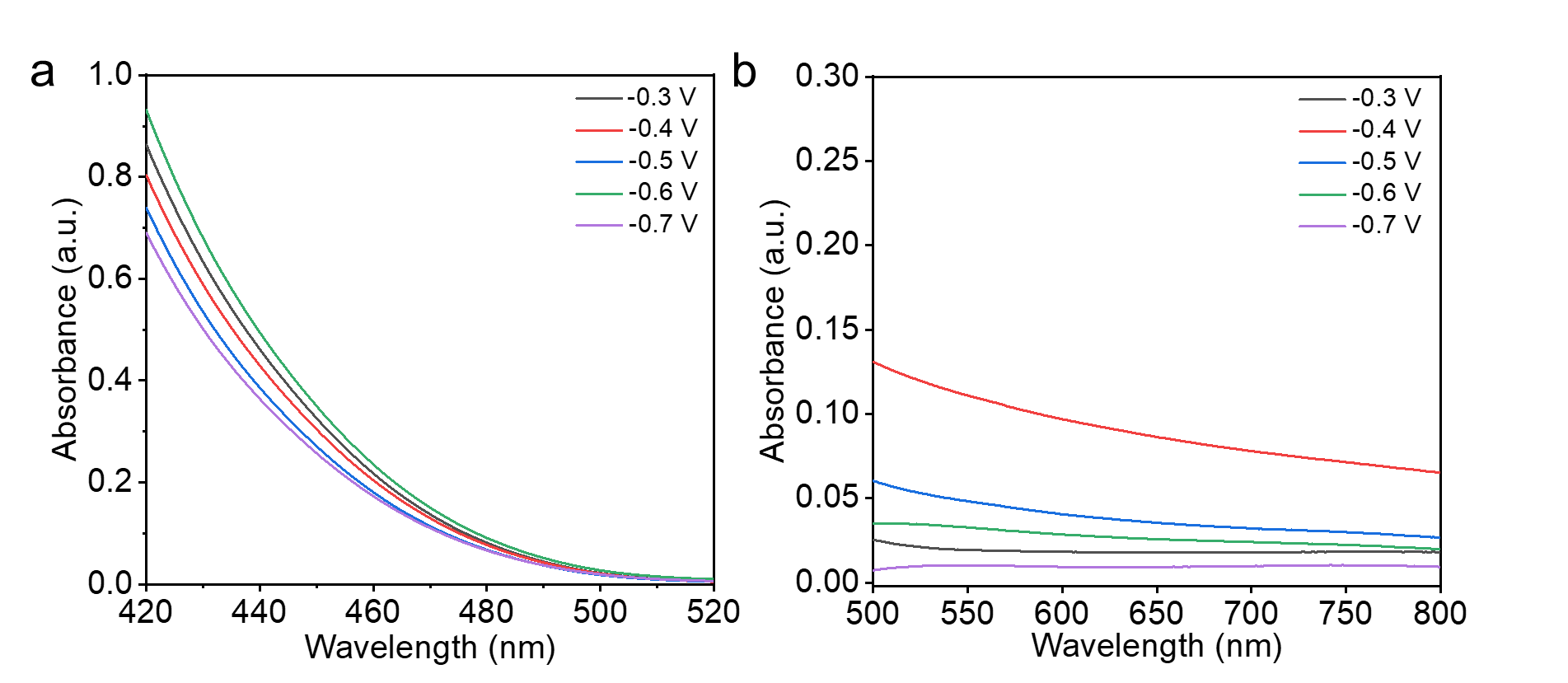


Figure S27. UV-Vis absorption spectra of N_2_H_4_ (a) and NH_2_OH (b) detected in the electrolyte after 1h of electrolysis over S-Cu@Co/C in H-cell.

There were no N_2_H_4_ and NH_2_OH in the electrolyte after 1h of electrolysis on the S-Cu@Co/C in the H-cell.


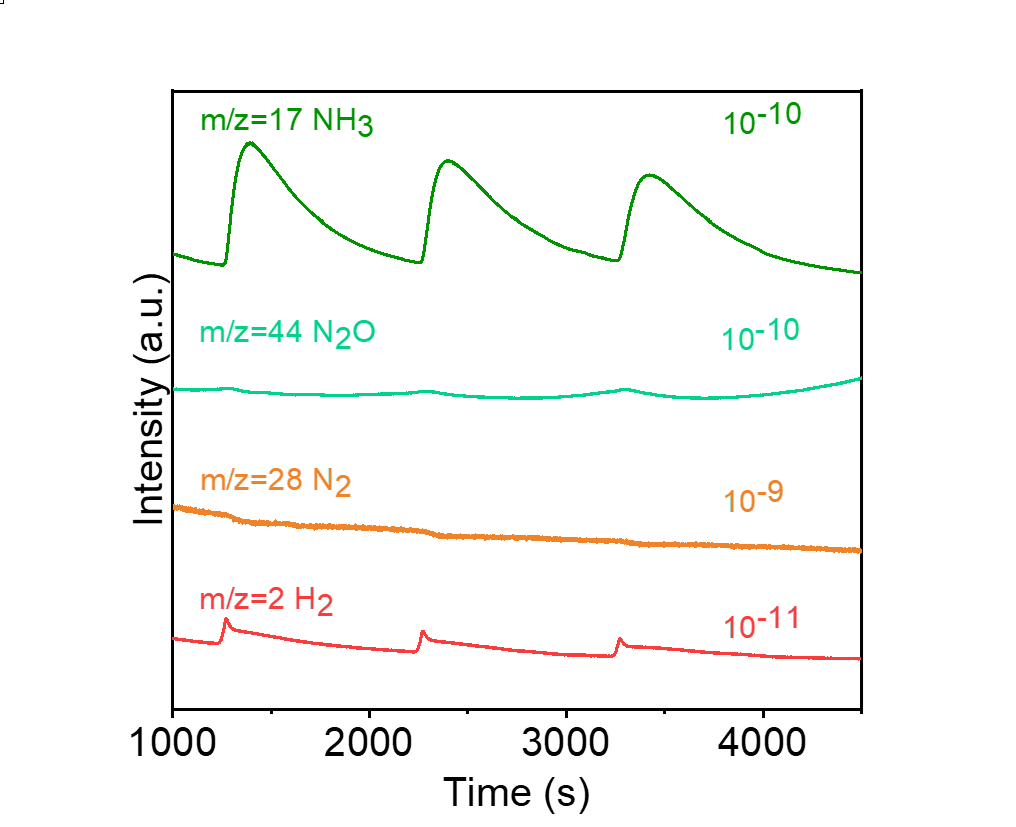


Figure S28. Online electrochemical differential mass spectrometry signals of gas products during the NORR on Cu@Co/C.

The signal of H_2_ in the DEMS of Cu@Co/C was much higher than that of S-Cu@Co/C, and there was also no N_2_O and N_2_ generated.


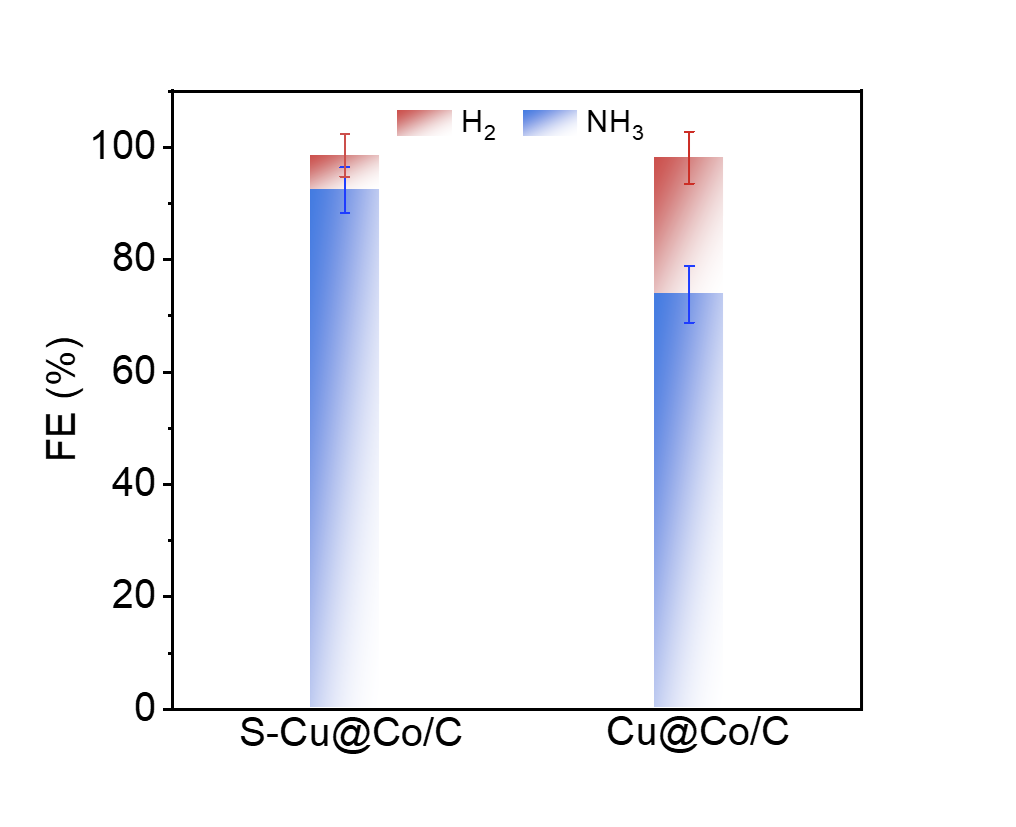


Figure S29. FE of NH_3_ and H_2_ over S-Cu@Co/C and Cu@Co/C at -0.6 V vs. RHE in H-cell.

The results show that hydrogen production on S-Cu@Co/C is significantly less than on Cu@Co/C.


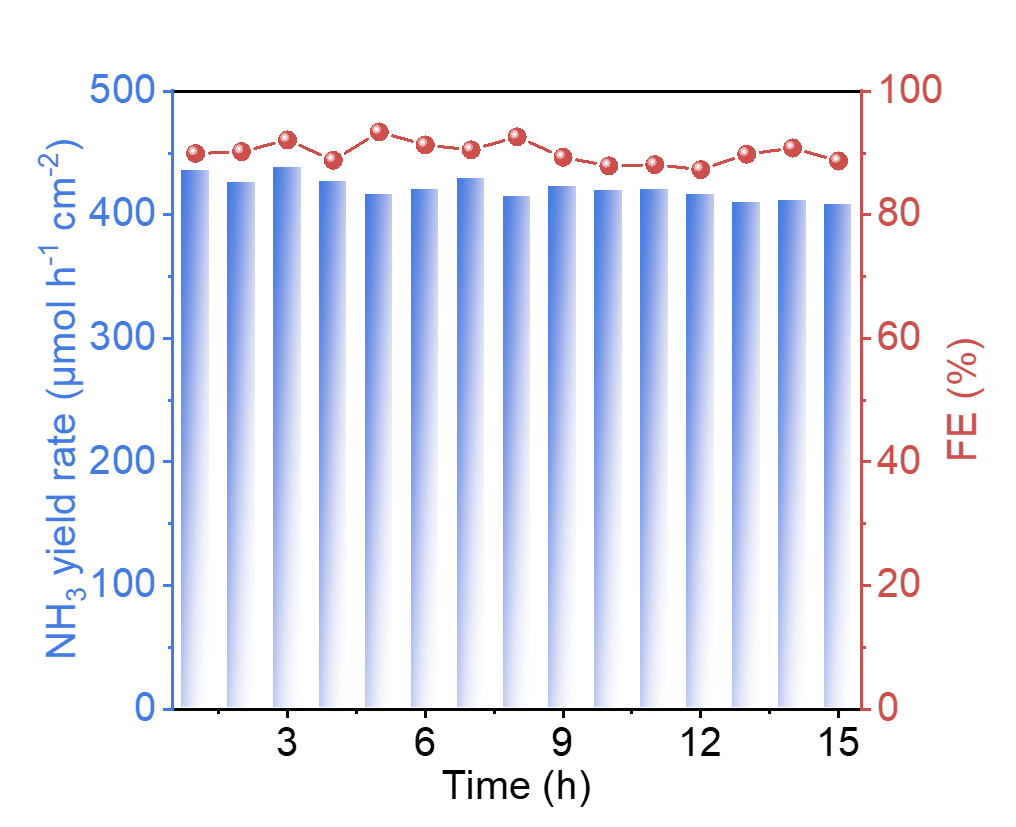


Figure S30. The NORR cycle test on S-Cu@Co/C at -0.6 V vs. RHE.

There was no noticeable decline in NH_3_ yield rate and Faradaic efficiency after fifteen consecutive electrolysis tests on S-Cu@Co/C.


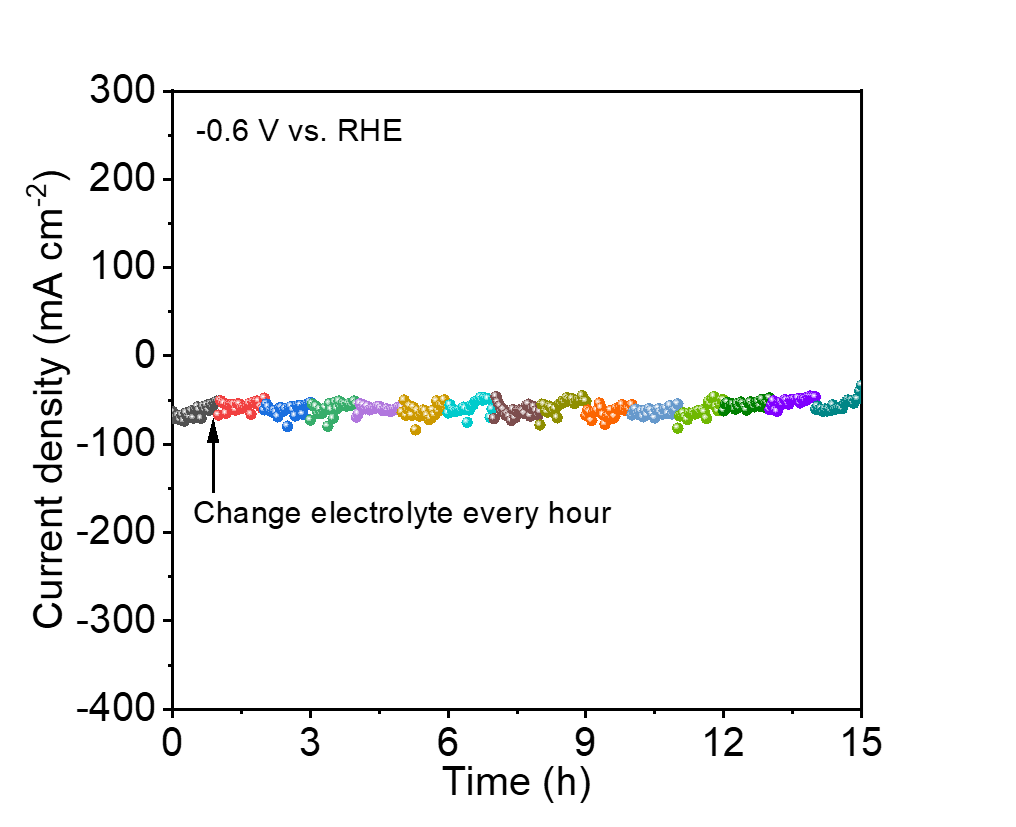


Figure S31. CA curves for S-Cu@Co/C in NO-saturated PBS electrolyte over 1 h electrolysis for fifteen cycles at -0.6 V vs. RHE.


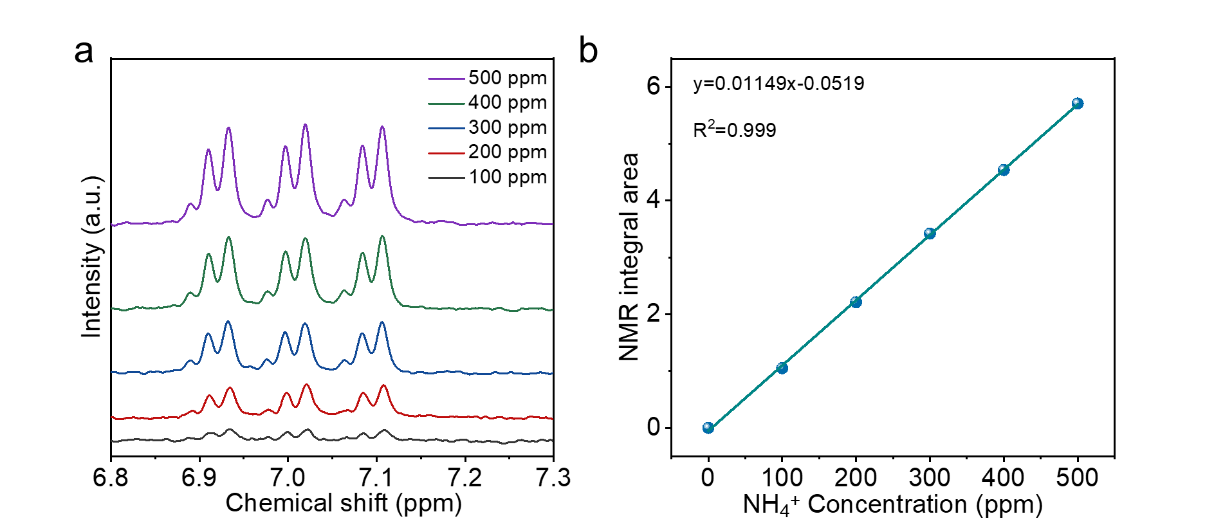


Figure S32. (a) ^1^H NMR spectra of standard NH_4_Cl solution. (b) Corresponding calibration curve of NMR integral area (NH_4_^+^-N/C_4_H_4_O_4_) against N (NH_4_^+^) concentration.

The concentration of NH_4_^+^ can be quantitatively determined by ^1^H-NMR. The NMR with internal standards (maleic acid, C_4_H_4_O_4_). The triple peaks were assigned to NH_4_^+^. The fitting curve obtains a good linear relationship between the NMR integral area and the NH_4_^+^ concentration.


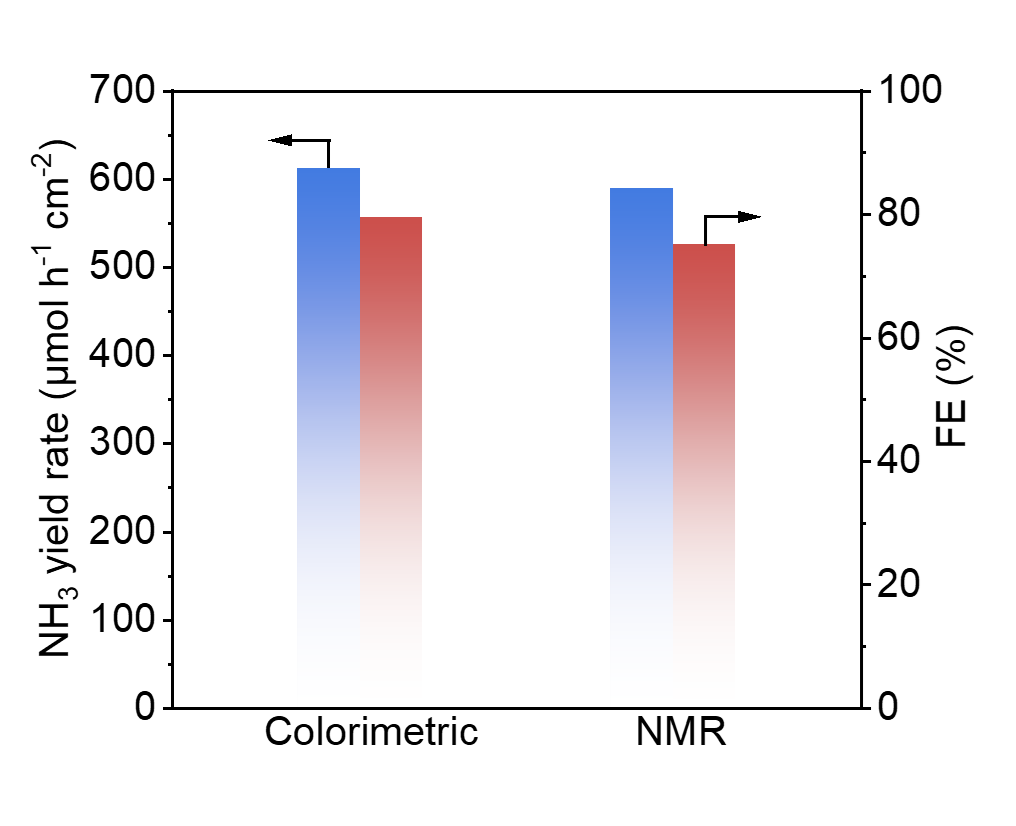


Figure S33. Comparison of NH_3_ yield and FE between the NMR and colorimetric methods on S-Cu@Co/C.

The quantitative NH_3_ yield rate and FE determined by ^1^H-NMR and colorimetric methods are similar.


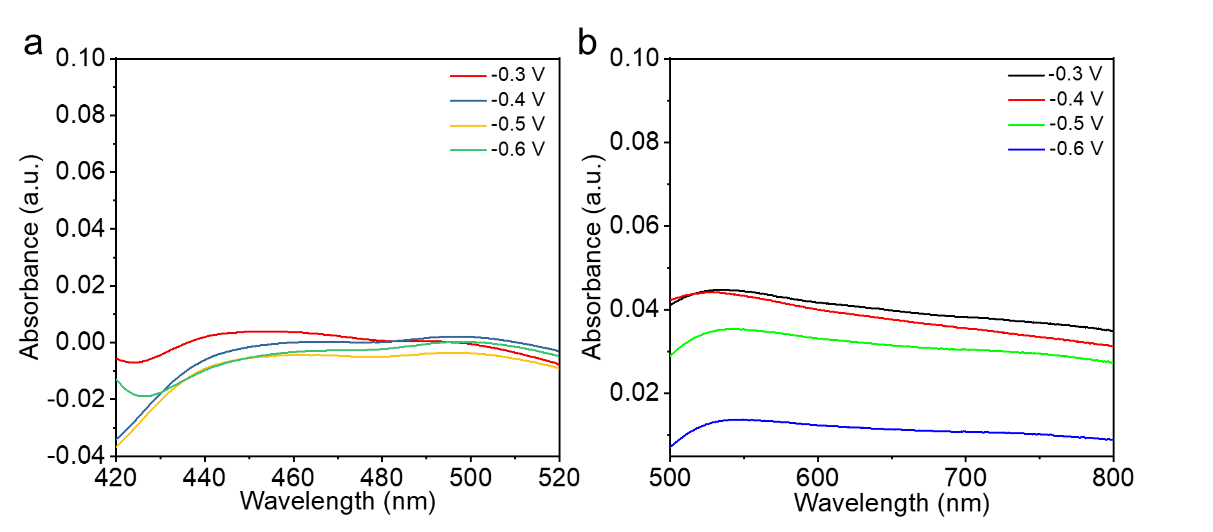


Figure S34. (a) UV-Vis absorption spectra of N_2_H_4_ and (b) NH_2_OH detected in the electrolyte after 1h of electrolysis over S-Cu@Co/C in flow cell.

There were no N_2_H_4_ and NH_2_OH in the electrolyte after 1h of electrolysis on the S-Cu@Co/C in the flow cell.


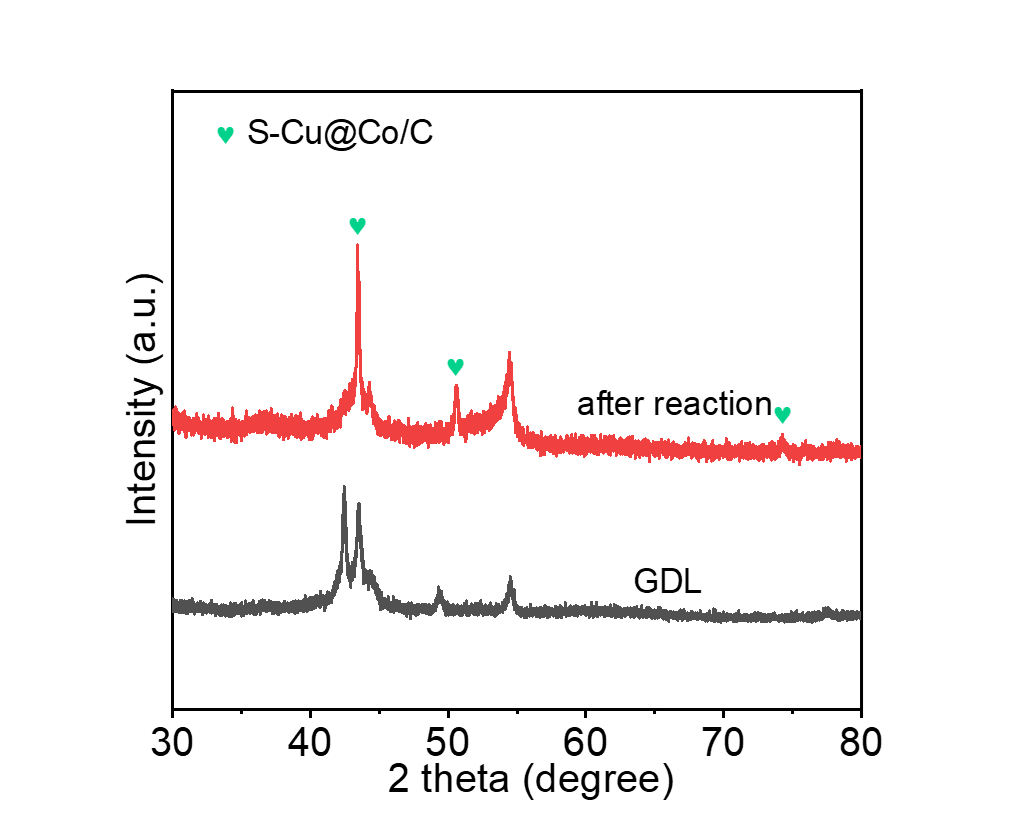


Figure S35. The XRD pattern of S-Cu@Co/C after stability test in the flow cell.

After the stability test in the flow cell, no significant destruction of the S-Cu@Co/C structure was observed.


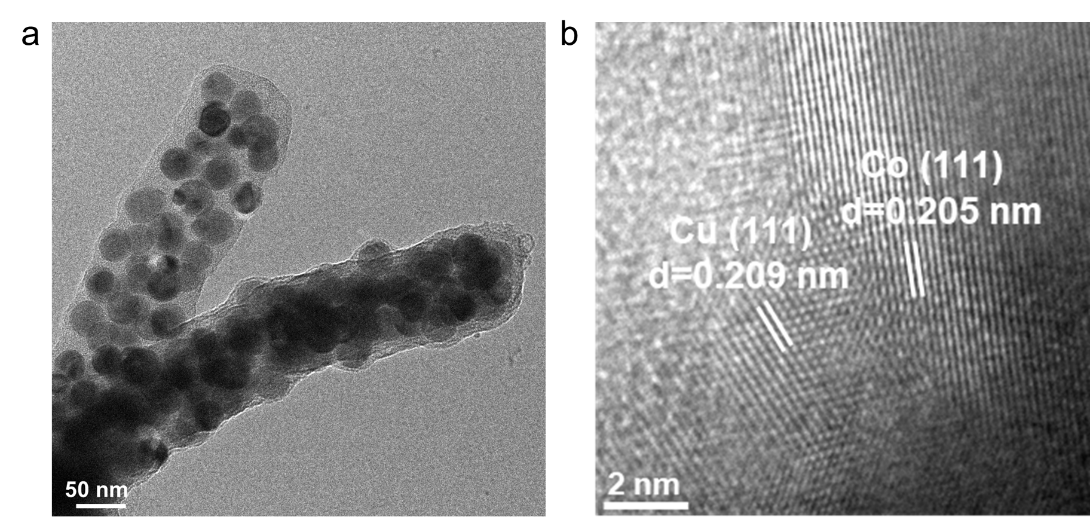


Figure S36. The HRTEM images of S-Cu@Co/C after stability test in the flow cell.


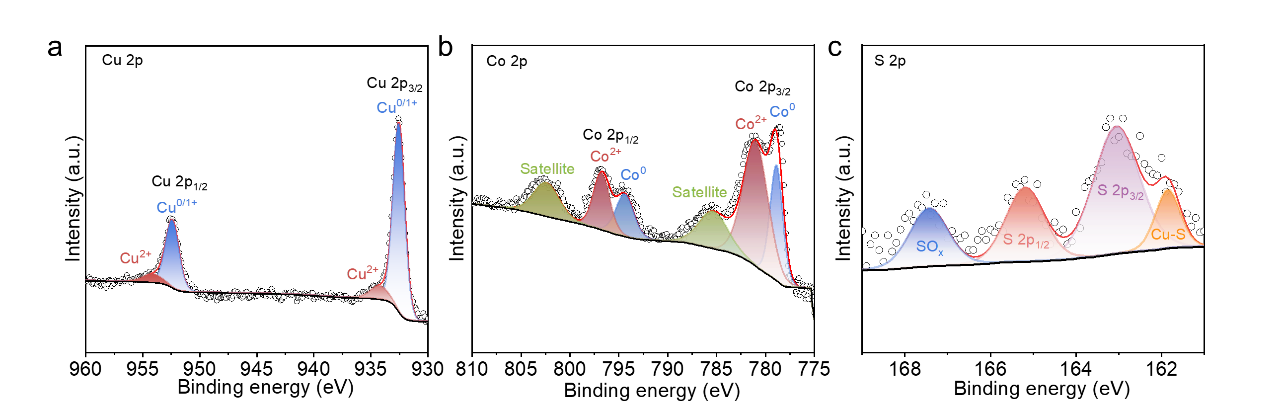


Figure S37. The XPS of S-Cu@Co/C after stability test in the flow cell.

After the stability test in the flow cell, no significant change in the surface valence state of S-Cu@Co/C.


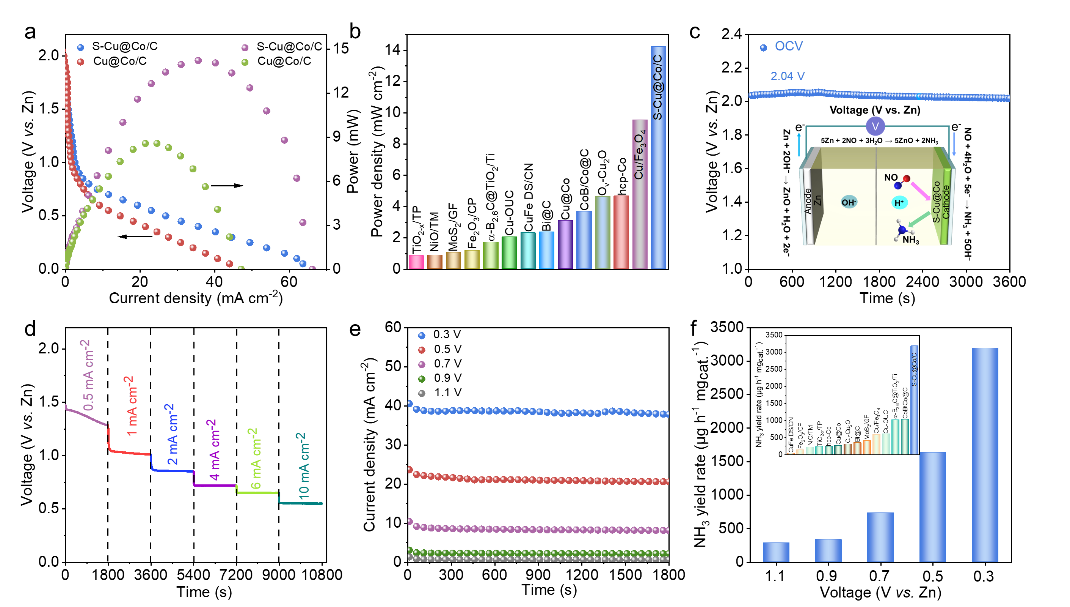


Figure S38. Zn-NO battery. (a) Polarization and power density curves of the S-Cu@Co/C and Cu@Co/C-based Zn–NO batteries. (b) Comparison of the peak power density of S-Cu@Co/C-based Zn–NO battery with the reported metal–NO battery. (c) OCV of the assembled Zn–NO battery with the S-Cu@Co/C cathode. The inset is a schematic illustration of the Zn–NO battery. (d) Discharging tests at various current densities. (e) Discharge current densities at different voltages. (f) NH_3_ yield rate at varied discharge voltages. The inset compares the NH_3_ yield rate in the S-Cu@Co/C-NO battery with the reported metal-NO batteries.

Benefitting from the outstanding electrocatalytic NORR performance of S-Cu@Co/C, an aqueous Zn-NO battery was assembled with S-Cu@Co as the cathode and the Zn plate as the anode. Figure S38a shows that the battery assembled with S-Cu@Co/C presents a power density of 14.23 mW cm^−2^, which is higher than that of Cu@Co/C and outperforms the currently reported metal-NO batteries (Figure S38b). Figure S38c shows the device displayed a high open-circuit voltage of 2.04 V vs. Zn. The discharging result shows that stable voltage outputs can be achieved when increasing the current density from 0.5 to 10 mA cm^−2^ (Figure S38d). The highest NH_3_ yield rate of 3192.6 μg h^-1^ mg_cat._^-1^ was achieved at 0.3 V vs. Zn under different voltages (Figure S38e-f), which is higher than the reported metal-NO batteries (Table S5).


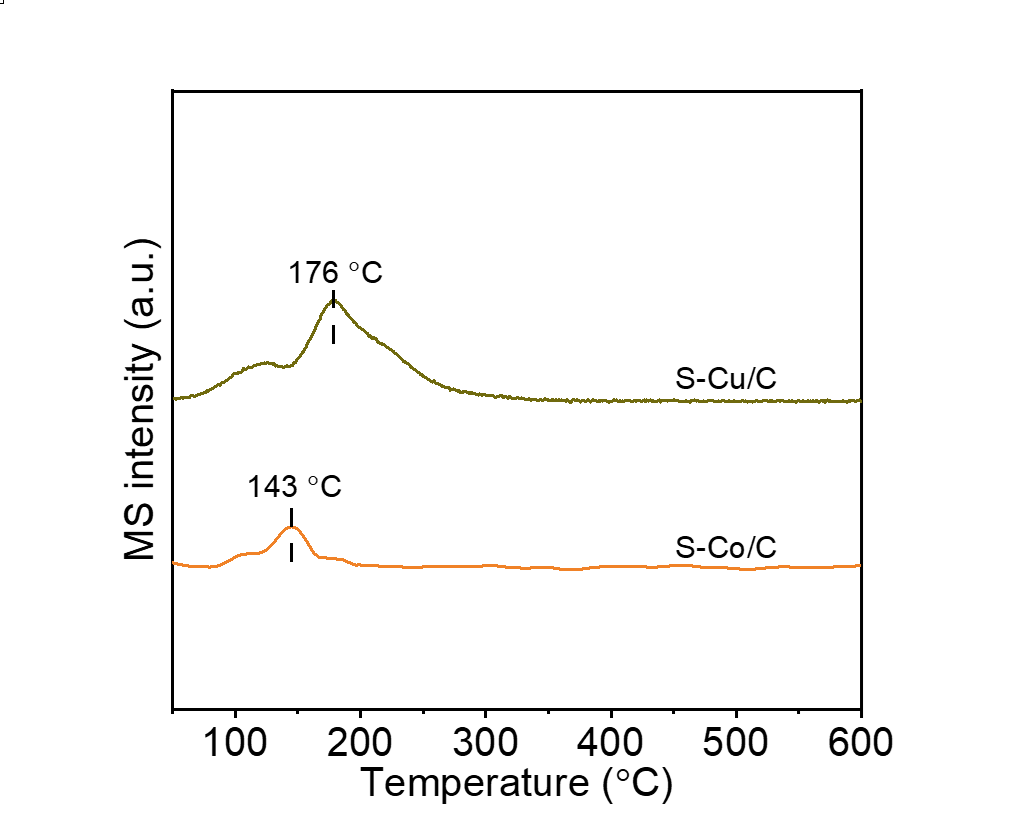


Figure S39. NO-TPD spectra of the S-Cu/C and S-Co/C.

The S-Cu/C exhibits a stronger NO adsorption than S-Co/C, implying that NO prefers to adsorb on Cu sites.


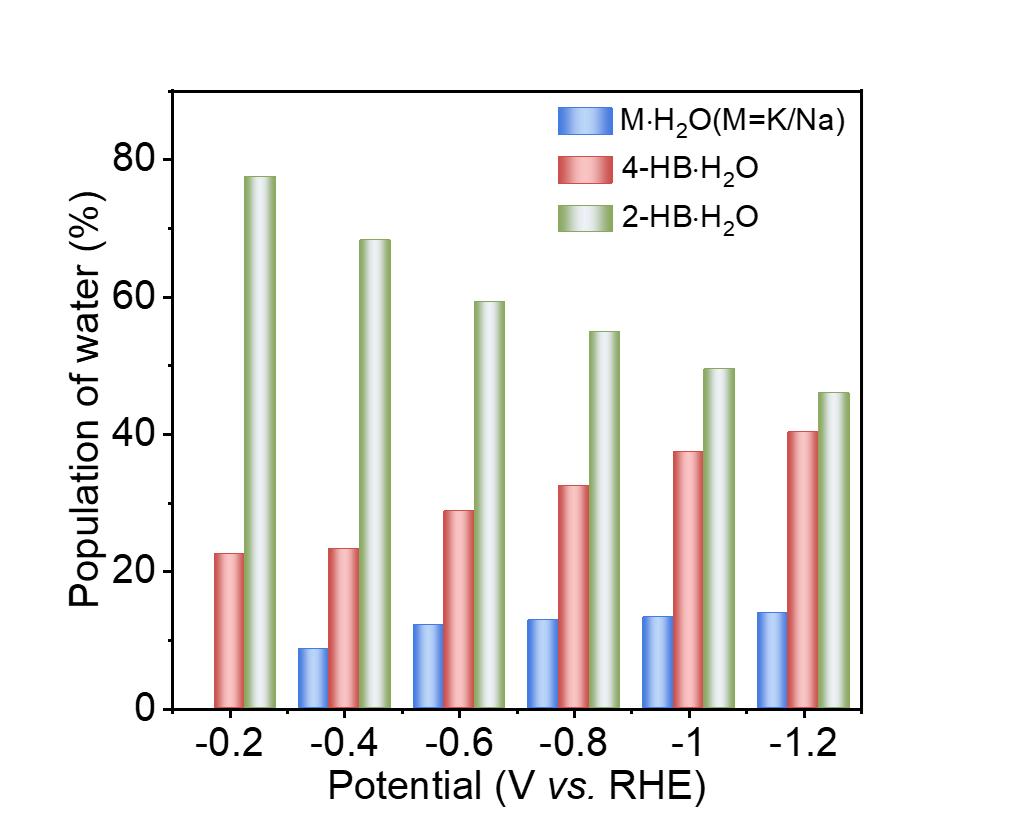


Figure S40. The potential-dependent population of interfacial water from in-situ Raman spectra fitting of Cu@Co/C.

Figure S40 shows that the proportion of K/Na·H_2_O on the Cu@Co/C surface was less than S-Cu@Co/C.


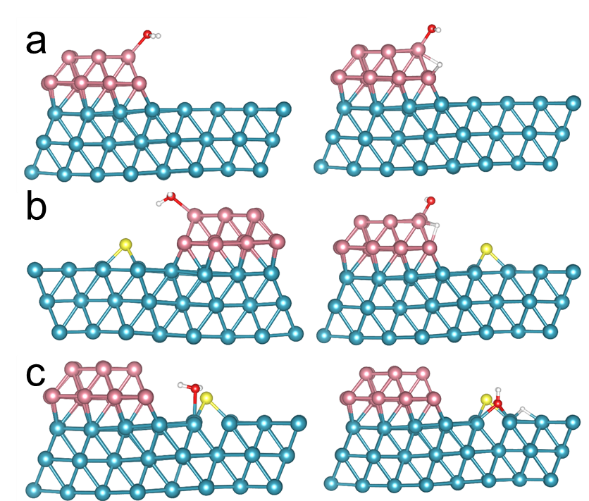


Figure S41. Geometrically optimized structural models for water dissociation calculations on Cu and Co sites in Cu@Co/C and S-Cu@Co/C.


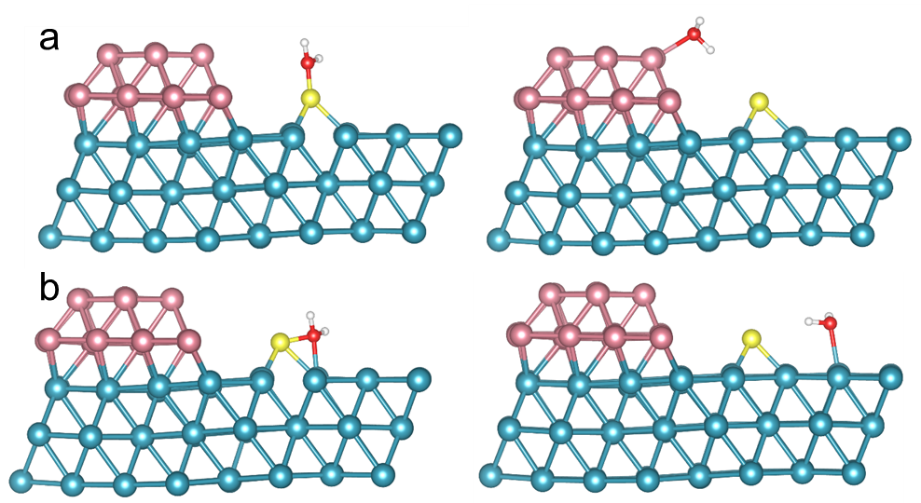


Figure S42. Geometrically optimized structural models for water dissociation calculations on S sites in S-Cu@Co/C.

The calculation results show that water dissociation cannot occur at the sulfur site.


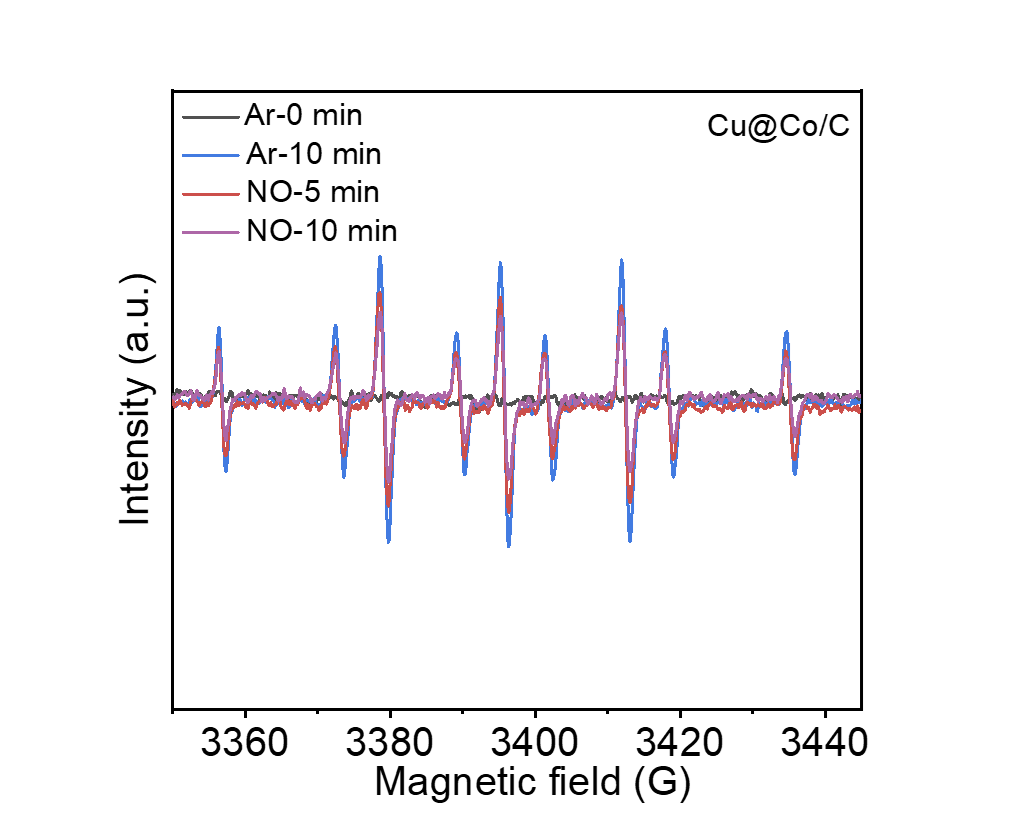


Figure S43. Quasi-in situ electrochemical EPR signals for Cu@Co/C in PBS electrolyte at -0.6 V vs. RHE.

When switched from Ar to NO, the decrease rate of the ⸱H on Cu@Co is slower compared to S-Cu@Co/C.


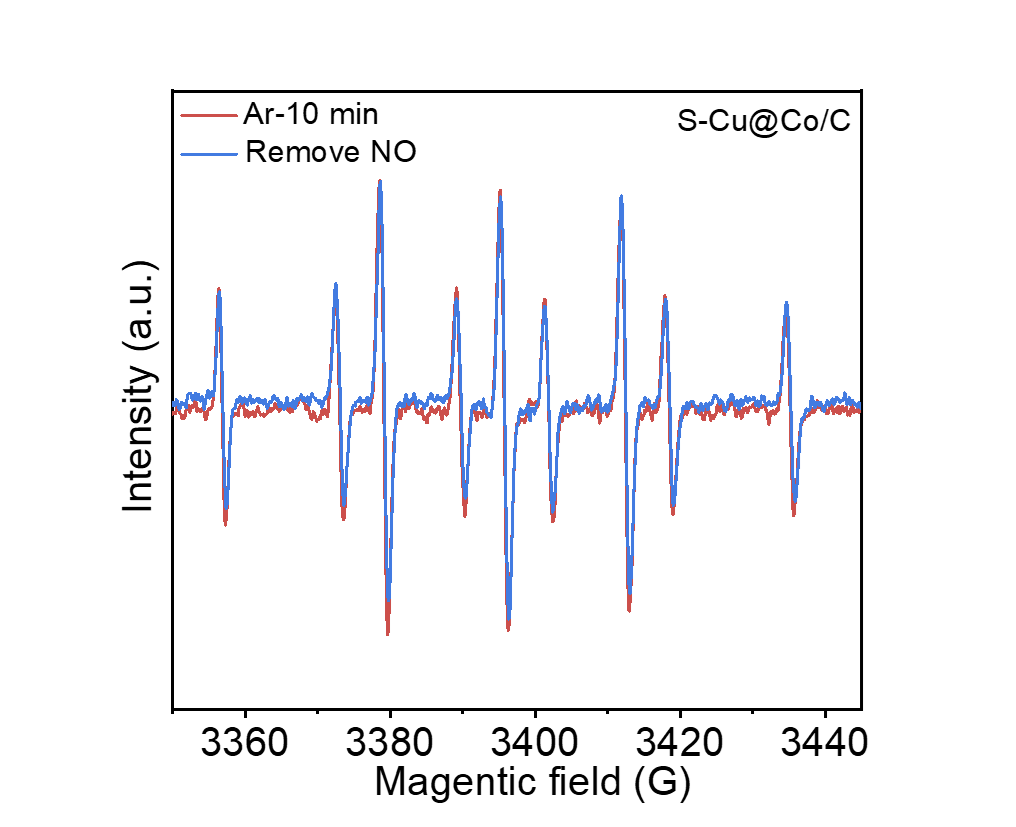


Figure S44. Quasi-in situ electrochemical EPR signals for S-Cu@Co/C in PBS electrolyte at -0.6 V vs. RHE.

When switching to Ar again, the ⸱H signal will be restored, suggesting that protons are essential for the reduction of NO.


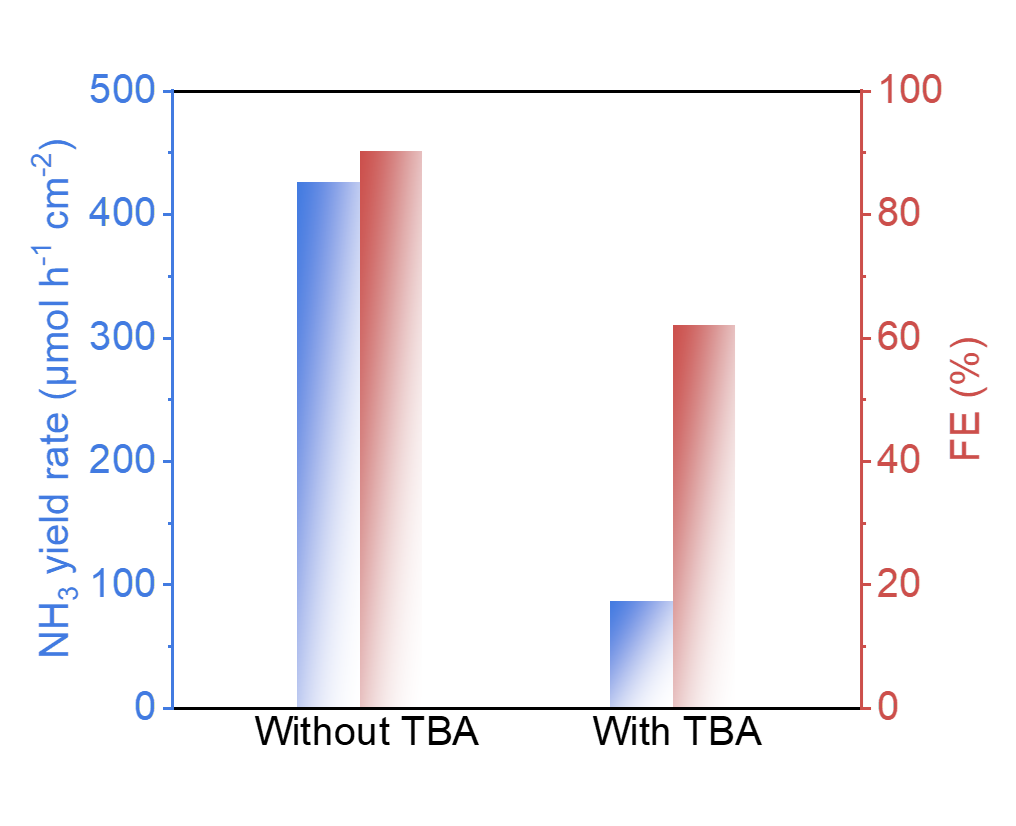


Figure S45. The NH_3_ yield rate and FE for the NORR over S-Cu@Co/C with and without TBA as *H scavenging.

The NH_3_ yield rate and FE of the CuNi@BCN were dramatically decreased to 85.74 μmol cm^-2^ h^-1^ and 61.98%, confirming the crucial role of hydrogen in the NORR process.


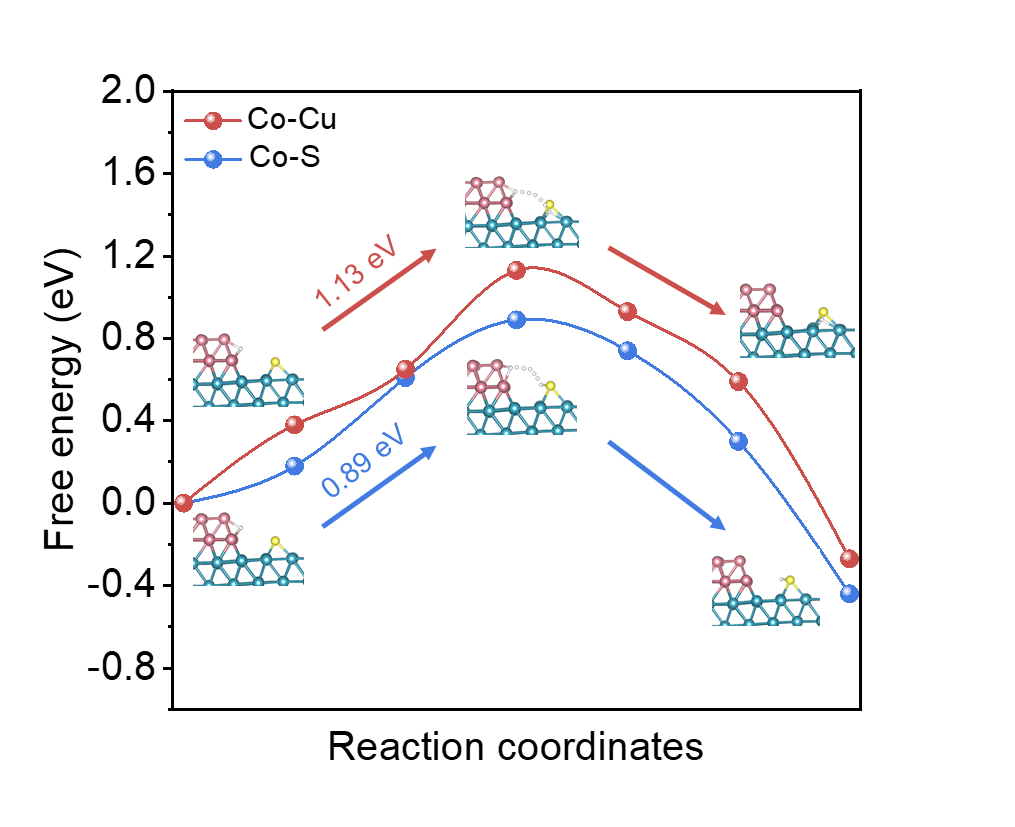


Figure S46. The Gibbs free energy diagram for proton transfer from the Co site to the Cu and sulfur site.


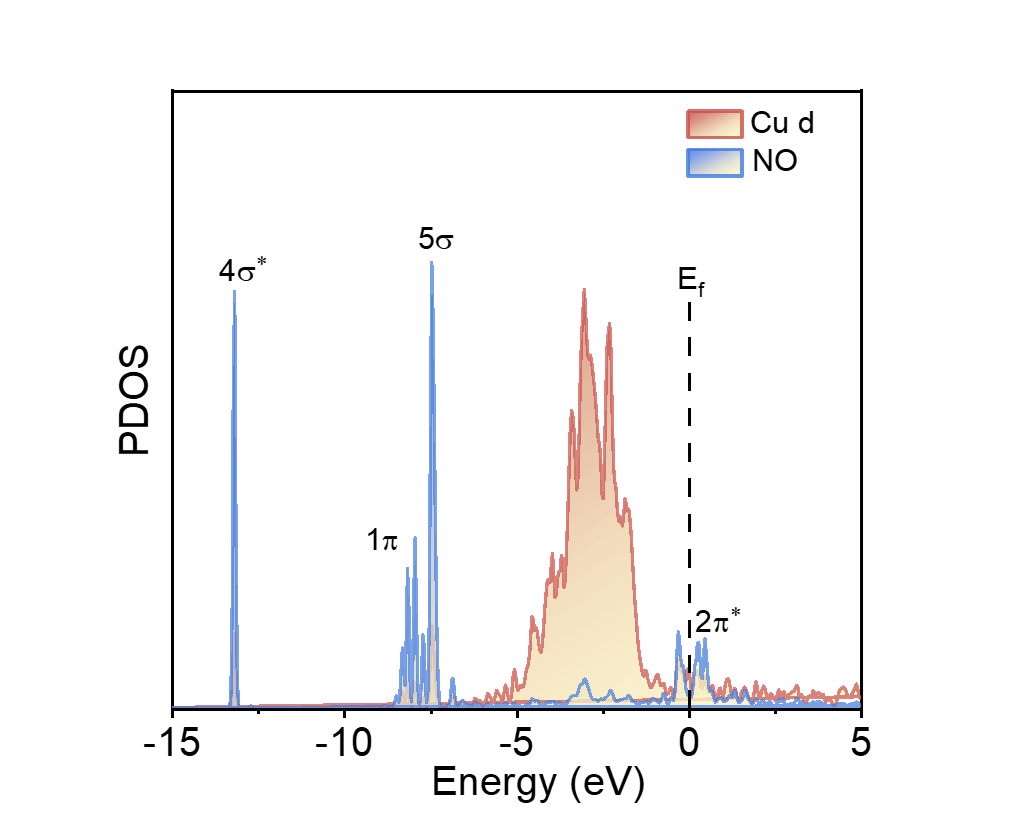


Figure S47. PDOS of *NO on S-Cu@Co/C.

The PDOS calculation revealed a strong electronic interaction between the Cu and *NO.


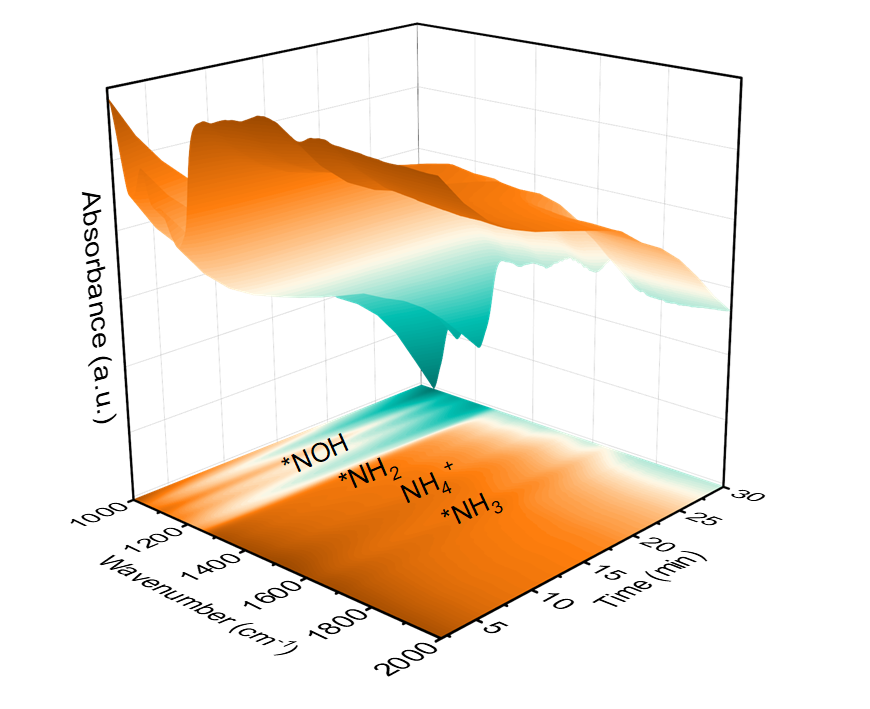


Figure S48. Operando ATR-IRAS spectra of electrocatalytic NORR under −0.6 V vs. RHE over Cu@Co/C catalyst.

Although similar intermediates and products were monitored on Cu@Co/C, the intensity of the peaks was significantly weakened compared to S-Cu@Co/C.


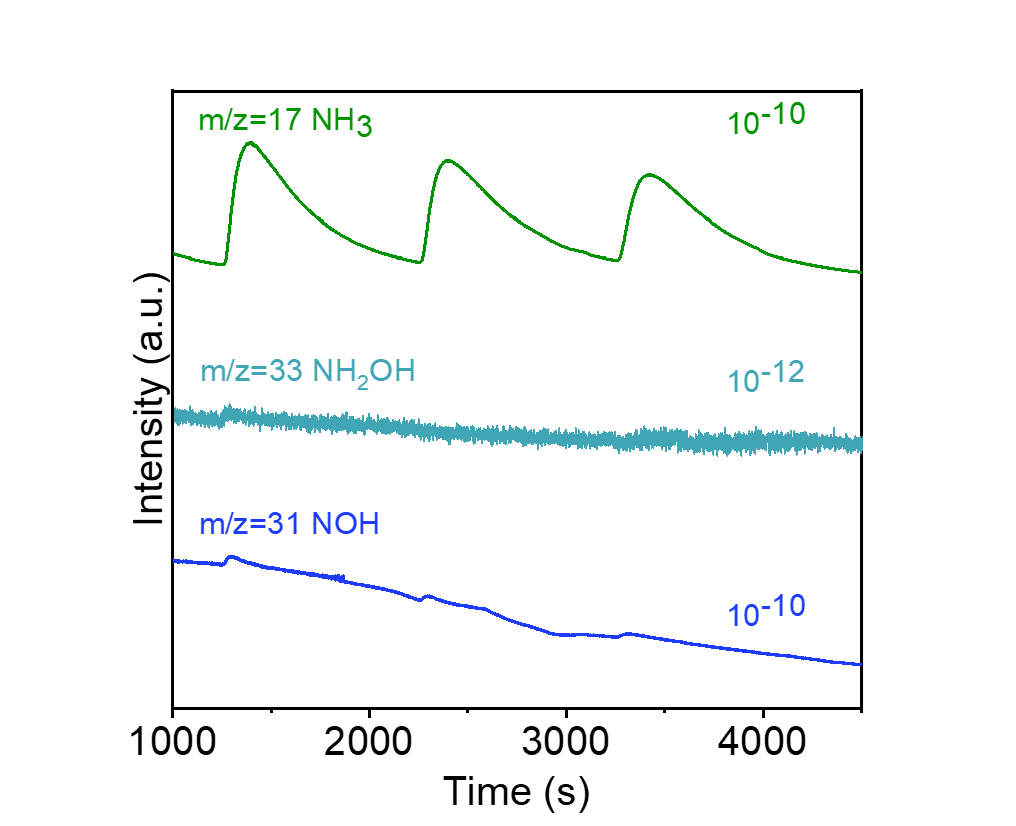


Figure S49. Online electrochemical differential mass spectrometry signals of products during the NORR on Cu@Co/C.

The signal intensity of the products and intermediates on Cu@Co/C is lower than that of S-Cu@Co/C during the NORR.


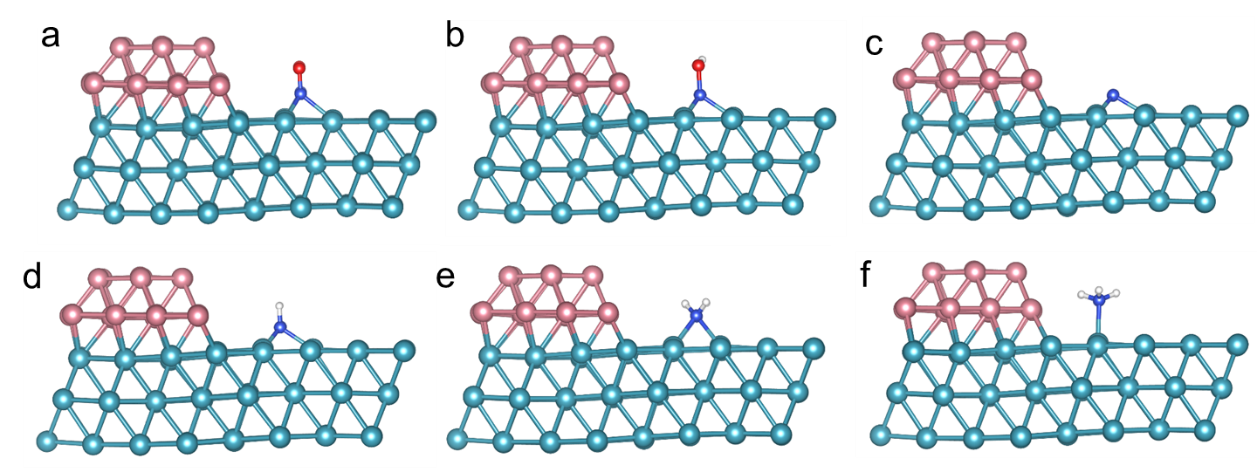


Figure S50. The diagram of the adsorption of reaction intermediates of NORR over Cu@Co/C after structural optimization. (a) to (f) correspond to *NO, *NOH, *N, *NH, *NH_2_ and *NH_3_, respectively.

**Table S1.** ICP-OES measurement results for S-Cu@Co/C and Cu@Co/C.

| Sample | Cu (W %) | Co (W %) | S (W %) |
| --- | --- | --- | --- |
| S-Cu@Co/C  Cu@Co/C | 51.8  55.6 | 12.4  12.9 | 0.31  / |

**Table S2**. EXAFS fitting results of the S-Cu@Co/C.

| Sample | Shell | CN | R(Å) | ΔE_0_(eV) | Δσ^2^*10^-3^(Å^2^) | R-factor |
| --- | --- | --- | --- | --- | --- | --- |
| Cu  Co | Cu-Cu  Cu-S  Co-Co | 10.6±2  0.5±0.3  9.3±1.9 | 2.53±0.01  2.11±0.01  2.49±0.01 | 2.6  3.7  -7.4 | 8.4  16.6  5.7 | 0.0045  0.0089 |

CN is the coordination number; R is the distance between absorber and backscatter atoms; σ^2^ is the Debye-Waller factor to account for both thermal and structural disorders; ΔE_0_ is the inner potential correction (edge-energy shift); R factor indicates the goodness of the fit.

**Table S3.** Evaluating intrinsic catalytic activity using mass activity and specific activity.

|  | Yield rate (μmol cm^-2^ h^-1^) | Mass activity (mol h^-1^ g_cat_^-1^) | Specific activity (mol m^-2^) |
| --- | --- | --- | --- |
| H-cell  Flow cell | 439.73  655.3 | 0.440  0.655 | 0.00272  0.00404 |

**Table S4**. Comparison of NH_3_ yield and FE of S-Cu@Co/C with reported NORR electrocatalysts.

| Catalysts | Electrolyte | *P*_NO_  (vol.%) | Cell | Potential (vs. RHE) | NH_3_ yield  (µmol h^−1^ cm^−2)^ | FE (%) | Ref. |
| --- | --- | --- | --- | --- | --- | --- | --- |
| S-Cu@Co/C  S-Cu@Co/C  Cu (111)  Cu/Fe_3_O_4_  Ni DHBT foam  Ru/C  hcp-Co  bcc RuGa-2 IMCs  CoB/Co@C  Nb-SA/BNC  Sb_1_/a-MoO_3_  In_1_/a-MoO_3_  a-B_2.6_C@TiO_2_/Ti  CuFe DS/NC  MoS_2_/GF  TiO_2−_*_x_*/TP  Ru-LCN  Cu@Co  Ag  Nanostructure  Cu_2_O@CoMn_2_O_4_ | PBS  PBS  0.1 M KOH  0.5 M Na_2_SO_4_  1 M KOH  1 M KOH  0.1 M Na_2_SO_4_  0.1 M K_2_SO_4_  0.5 M Na_2_SO_4_  0.1 M HCl  0.5 M Na_2_SO_4_  0.5 M Na_2_SO_4_  0.1 M Na_2_SO_4_  +0.5mM Fe^2+^-EDTA  0.1 M Na_2_SO_4_  0.1 M HCl  +0.5mM Fe(II)SB  0.2 M PBS  0.5 M Na_2_SO_4_  0.1 M Na_2_SO_4_  0.5 M PBS+  0.5mM EDTA  0.1 M Na_2_SO_4_ | 10  10  99  99  100  5.2  100  20  99  99  100  99  10  99  10  10  1  1  1  99 | H–cell  Flow–cell  H–cell  H–cell  MEA  MEA  H–cell  H–cell  H-cell  Flow-cell  H-cell  H–cell  H–cell  H–cell  H–cell  H–cell  Flow–cell  H–cell  H–cell  H–cell | -0.6 V  -0.5 V  -0.59 V  -0.5 V  /  /  -0.6 V  -0.2 V  -0.6 V  -0.6 V  -0.6 V  -0.6 V  -0.9 V  -0.6 V  -0.7 V  -0.7 V  -0.2 V  -0.5 V  -0.165 V  -0.8 V | 439.73  655.3  187.5  347.5  582  510  439.5  320.6  315.4  296.5  273.5  242.6  216.4  112.5  99.6  72.54  45  36.9  36.9  6.3 | 92.4  77.4  93.19  95.8  78  57  72.58  72.3  70  77  91.7  92.8  87.6  90  76.6  66  65.9  76.5  100  75.1 | This work  This work  ^[5]^  ^[6]^  ^[7]^  ^[8]^  ^[9]^  ^[10]^  ^[11]^  ^[12]^  ^[13]^  ^[14]^  ^[15]^  ^[16]^  ^[17]^  ^[18]^  ^[19]^  ^[20]^  ^[21]^  ^[22]^ |

**Table S5**. Comparison of NH_3_ yield and peak power density of S-Cu@Co/C-NO battery with reported metal-NO battery systems.

| Catalysts | Battery systems | Power density | NH_3_ yield | Ref. |
| --- | --- | --- | --- | --- |
| S-Cu@Co/C  Cu/Fe_3_O_4_  hcp-Co  O_v_-Cu_2_O  CoB/Co@C  Cu@Co  Bi@C  CuFe DS/NC  Cu-OUC  a-B_2.6_C@TiO_2_/Ti  Fe_2_O_3_/CP  MoS_2_/GF  NiO/TM  TiO_2−_*_x_*/TP | metal-NO  metal-NO  metal-NO  metal-NO  metal-NO  metal-NO  metal-NO  metal-NO  metal-NO  metal-NO  metal-NO  metal-NO  metal-NO  metal-NO | 14.23 mW cm^-2^  9.53 mW cm^−2^  4.66 mW cm^-2^  4.62 mW cm^-2^  3.68 mW cm^–2^  3.08 mW cm^-2^  2.35 mW cm^-2^  2.30 mW cm^−2^  2.04 mW cm^−2^  1.7 mW cm^−2^  1.18 mW cm^−2^  1.04 mW cm^-2^  0.88 mW cm^−2^  0.84 mW cm^−2^ | 3192.6 µg h^−1^ mg^−1^  595.7 µg h^−1^ cm^−2^  247.8 µg h^−1^ mg^−1^  308.0 µg h^−1^ mg^−1^  1035.3 μg h^–1^ cm^–1^  273.37 µg h^−1^ cm^−2^  355.6 µg h^−1^ mg^−1^  45.52 µg h^−1^ mg^−1^  616.9 µg h^−1^ mg^−1^  1023 µg h^−1^ mg^−1^  145.28 μg h^−1^ mg^−1^  411.8 μg h^−1^ mg_._^−1^  228 μg h^−1^ cm^−2^  241.7 µg h^−1^ cm^−2^ | This work  ^[6]^  ^[23]^  ^[24]^  ^[11]^  ^[20]^  ^[25]^  ^[16]^  ^[26]^  ^[15]^  ^[27]^  ^[17]^  ^[28]^  ^[18]^ |

**References**

[1] a) W. Kohn, L. J. Sham, *Phys. Rev.* **1965**, *140*, A1133-A1138; b) P. Raybaud, J. Hafner, G. Kresse, S. Kasztelan, H. Toulhoat, *J. Catal.* **2000**, *189*, 129-146; c) G. Kresse, J. Furthmüller, *Phys. Rev. B* **1996**, *54*, 11169-11186.

[2] J. P. Perdew, K. Burke, M. Ernzerhof, *Phys. Rev. Lett.* **1996**, *77*, 3865-3868.

[3] a) P. E. Blöchl, *Phys. Rev. B* **1994**, *50*, 17953-17979; b) G. Kresse, D. Joubert, *Phys. Rev. B* **1999**, *59*, 1758-1775.

[4] a) S. Grimme, J. Antony, S. Ehrlich, H. Krieg, *J Chem Phys* **2010**, *132*, 154104; b) S. Grimme, S. Ehrlich, L. Goerigk, *J. Comput. Chem.* **2011**, *32*, 1456-1465.

[5] L. Xiao, S. Mou, W. Dai, W. Yang, Q. Cheng, S. Liu, F. Dong, *Angew. Chem. Int. Ed.* **2024**, *63*, e202319135.

[6] L. Liu, Z.-J. Zuo, Y. Du, T. Wu, J. Wu, J. Gao, T. Mu, Y.-C. Zhang, X.-D. Zhu, *J. Colloid Interface Sci.* **2025**, *691*, 137376.

[7] A. Singh-Morgan, K. Trösch, A. Weinfurter, M. Inniger, Y.-Z. Xu, V. Mougel, *Chem* **2025**, 102460.

[8] S. Bunea, M. Coppens, A. Urakawa, *ACS Catal.* **2023**, *13*, 11345-11351.

[9] D. Wang, Z. W. Chen, K. Gu, C. Chen, Y. Liu, X. Wei, C. V. Singh, S. Wang, *J. Am. Chem. Soc.* **2023**, *145*, 6899-6904.

[10] H. Zhang, Y. Li, C. Cheng, J. Zhou, P. Yin, H. Wu, Z. Liang, J. Zhang, Q. Yun, A.-L. Wang, L. Zhu, B. Zhang, W. Cao, X. Meng, J. Xia, Y. Yu, Q. Lu, *Angew. Chem. Int. Ed.* **2023**, *62*, e202213351.

[11] B. Wu, L. Huang, L. Yan, H. Gang, Y. Cao, D. Wei, H. Wang, Z. Guo, W. Zhang, *Nano Lett.* **2023**, *23*, 7120-7128.

[12] X. Peng, Y. Mi, H. Bao, Y. Liu, D. Qi, Y. Qiu, L. Zhuo, S. Zhao, J. Sun, X. Tang, J. Luo, X. Liu, *Nano Energy* **2020**, *78*, 105321.

[13] K. Chen, Y. Zhang, J. Xiang, X. Zhao, X. Li, K. Chu, *ACS Energy Lett.* **2023**, *8*, 1281-1288.

[14] K. Chen, N. Zhang, F. Wang, J. Kang, K. Chu, *J. Mater. Chem. A* **2023**, *11*, 6814-6819.

[15] J. Liang, P. Liu, Q. Li, T. Li, L. Yue, Y. Luo, Q. Liu, N. Li, B. Tang, A. A. Alshehri, I. Shakir, P. O. Agboola, C. Sun, X. Sun, *Angew. Chem. Int. Ed.* **2022**, *61*, e202202087.

[16] D. Wang, X. Zhu, X. Tu, X. Zhang, C. Chen, X. Wei, Y. Li, S. Wang, *Adv. Mater.* **2023**, *35*, 2304646.

[17] L. Zhang, J. Liang, Y. Wang, T. Mou, Y. Lin, L. Yue, T. Li, Q. Liu, Y. Luo, N. Li, B. Tang, Y. Liu, S. Gao, A. A. Alshehri, X. Guo, D. Ma, X. Sun, *Angew. Chem. Int. Ed.* **2021**, *60*, 25263-25268.

[18] Z. Li, Q. Zhou, J. Liang, L. Zhang, X. Fan, D. Zhao, Z. Cai, J. Li, D. Zheng, X. He, Y. Luo, Y. Wang, B. Ying, H. Yan, S. Sun, J. Zhang, A. A. Alshehri, F. Gong, Y. Zheng, X. Sun, *Small* **2023**, *19*, 2300291.

[19] Y. Li, C. Cheng, S. Han, Y. Huang, X. Du, B. Zhang, Y. Yu, *ACS Energy Lett.* **2022**, *7*, 1187-1194.

[20] Z. Wu, Y. Liu, D. Wang, Y. Zhang, K. Gu, Z. He, L. Liu, H. Liu, J. Fan, C. Chen, S. Wang, *Adv. Mater.* **2024**, *36*, 2309470.

[21] D. Kim, D. Shin, J. Heo, H. Lim, J.-A. Lim, H. M. Jeong, B.-S. Kim, I. Heo, I. Oh, B. Lee, M. Sharma, H. Lim, H. Kim, Y. Kwon, *ACS Energy Lett.* **2020**, *5*, 3647-3656.

[22] C. Bai, S. Fan, X. Li, Z. Niu, J. Wang, Z. Liu, D. Zhang, *Adv. Funct. Mater.* **2022**, *32*, 2205569.

[23] D. Wang, Z.-W. Chen, K. Gu, C. Chen, Y. Liu, X. Wei, C. V. Singh, S. Wang, *J. Am. Chem. Soc.* **2023**, *145*, 6899-6904.

[24] S. Liu, L. Yan, Q. Chen, B. Wu, H. Zhu, S. Zhou, X. Wang, X. Zhao, C. Sun, Y. Li, L. Lei, Z. Li, Y. Hou, B. Yang, *Process Saf. Environ. Prot.* **2024**, *187*, 312-319.

[25] Q. Liu, Y. Lin, L. Yue, J. Liang, L. Zhang, T. Li, Y. Luo, M. Liu, J. You, A. A. Alshehri, Q. Kong, X. Sun, *Nano Res.* **2022**, *15*, 5032-5037.

[26] R. Luo, B.-J. Li, Z.-P. Wang, M.-G. Chen, G.-L. Zhuang, Q. Li, J.-P. Tong, W.-T. Wang, Y.-H. Fan, F. Shao, *JACS Au* **2024**, *4*, 3823-3832.

[27] J. Liang, H. Chen, T. Mou, L. Zhang, Y. Lin, L. Yue, Y. Luo, Q. Liu, N. Li, A. A. Alshehri, I. Shakir, P. O. Agboola, Y. Wang, B. Tang, D. Ma, X. Sun, *J. Mater. Chem. A* **2022**, *10*, 6454-6462.

[28] P. Liu, J. Liang, J. Wang, L. Zhang, J. Li, L. Yue, Y. Ren, T. Li, Y. Luo, N. Li, B. Tang, Q. Liu, A. M. Asiri, Q. Kong, X. Sun, *Chem. Commun.* **2021**, *57*, 13562-13565.
